# Biofilm formation and polar lipid biosynthesis in *Mycobacterium abscessus* are inhibited by naphthylmethylpiperazine

**DOI:** 10.1371/journal.pone.0311669

**Published:** 2024-11-12

**Authors:** Timilehin Faboro, Jaiyanth Daniel

**Affiliations:** Department of Biological Sciences, Purdue University Fort Wayne, Fort Wayne, IN, United States of America; The University of New Mexico School of Medicine, UNITED STATES OF AMERICA

## Abstract

*Mycobacterium abscessus* is a biofilm-forming, non-tuberculous mycobacterium that is highly resistant to antibiotics. Bacterial efflux pumps contribute to biofilm formation, export of biofilm-associated lipids and antibiotic tolerance. The Resistance Nodulation Cell Division (RND) and ATP-Binding Cassette (ABC) families of efflux pumps export lipids to the mycobacterial cell surface. 1-(1-naphthyl methyl)-piperazine (NMP) is a chemosensitizer that causes membrane destabilization and is an inhibitor of RND efflux pumps. The effects of NMP on biofilm formation and lipid metabolism in *M*. *abscessus* biofilms have not been investigated. Plumbagin (PLU) is an inhibitor of ABC efflux pumps that has not been studied for its effects on antibiotic tolerance in *M*. *abscessus* biofilms. In this study, we report that the efflux pump inhibitors NMP and PLU inhibit biofilm formation by 50% at sub-MIC levels. We show that NMP inhibits the incorporation of the radiolabeled long-chain fatty acid ^14^C-palmitate into glycopeptidolipids in cell surface lipids of log-phase *M*. *abscessus*. NMP also inhibits the utilization of the radiolabel in the biosynthesis of phosphatidylethanolamine in the cell surface and cellular lipids of *M*. *abscessus* cells in log-phase and in biofilms. Incorporation of the radiolabel into cardiolipin in the cellular lipids of *M*. *abscessus* biofilms was inhibited by NMP. The incorporation of ^14^C-acetate into cell surface phosphatidylethanolamine in log-phase and biofilm cells was also inhibited by NMP. Triacylglycerol biosynthesis using ^14^C-palmitate and ^14^C-acetate in cellular lipids of log-phase and biofilm cells was increased several folds by NMP. Efflux pump activity in *M*. *abscessus* cells was inhibited by 97% and 68% by NMP and PLU respectively. NMP and PLU caused 5-fold decreases in the minimum inhibitory concentrations of ciprofloxacin and clarithromycin against *M*. *abscessus*. Our results demonstrate that NMP and PLU affect important physiological processes in *M*. *abscessus* associated with its pathogenesis.

## 1. Introduction

*Mycobacterium abscessus* is a biofilm-forming, antibiotic-tolerant, non-tuberculous mycobacterium and causes lung and soft tissue infections [1]. The treatment for *M*. *abscessus* infections involves a combination of antibiotics that include ciprofloxacin (CIP), clarithromycin (CLA), amikacin (AMI) and cefoxitin (CEF) [2].

*M*. *abscessus* has been shown to be capable of persisting in macrophages and accumulating the storage lipid triacylglycerol [3]. We had demonstrated earlier that *Mycobacterium tuberculosis* imports fatty acids derived from the lipid bodies of human macrophages and utilizes them for the biosynthesis of triacylglycerol and polar lipids [4]. *M*. *abscessus* inside macrophages access macrophage lipid droplets that are an abundant source of exogenous fatty acids for lipid biosynthesis in the pathogen, as shown previously for *M*. *tuberculosis* [3–5]. The caseous granuloma could also provide a lipid-rich carbon source to *M*. *abscessus* [6, 7]. Polar lipids on the cell surface of non-tuberculous mycobacteria have been known to play vital roles in cell-to-cell interactions and glycopeptidolipids (GPL) have been extensively studied in *M*. *smegmatis*, *M*. *abscessus* and other non-tuberculous mycobacteria [8–10]. We showed recently that the non-tuberculous *Mycobacterium smegmatis* utilizes exogenously provided long-chain fatty acids in the biosynthesis of GPLs [11]. Recent reports on the metabolism of lipids in biofilms of *M*. *abscessus* and *Mycobacterium chelonae* used radiolabeled ^14^C-acetic acid [12, 13]. However, there have been no studies on the metabolic utilization of exogenously-derived long-chain fatty acids in the biosynthesis of cell surface and cellular polar lipids in *M*. *abscessus* biofilms.

Lipids constitute the most abundant component of the extracellular matrix of *M*. *abscessus* biofilms and GPLs and other polar lipids have been implicated in biofilm formation in *M*. *abscessus* and *M*. *smegmatis* [12, 14, 15]. Efflux pumps are associated with the export of extracellular polymeric substances and their inactivation by efflux pump inhibitors (EPIs) blocked bacterial biofilm formation [16]. Mycobacterial efflux pumps belonging to the resistance nodulation cell division (RND) and ATP binding cassette (ABC) families have been shown to be involved in the export of glycolipids to the cell envelope [17]. EPIs such as 1-(1-naphthylmethyl)-piperazine (NMP) and plumbagin (PLU) have been shown to inhibit efflux pumps belonging to the RND and ABC families, respectively [18–20]. NMP has been shown to inhibit RND family efflux pumps associated with fluoroquinolone tolerance and reduce biofilm formation in *Escherichia coli* [21, 22]. NMP acts as a chemosensitizer causing membrane destabilization in *Klebsiella pneumoniae* and inhibits biofilm formation and ABC transporter gene expression in *M*. *tuberculosis* [23, 24]. PLU has been reported to be a substrate and an inhibitor of the eukaryotic ABCG2 drug transporter [25]. However, the effects of either one of these EPIs on lipid biosynthesis during biofilm formation in *M*. *abscessus* have not been investigated.

*M*. *abscessus* forms biofilms in which the expression of efflux pumps is upregulated [26, 27]. It has been shown that mycobacteria use efflux pumps to develop antimicrobial tolerance [28–30]. EPIs block the activities of efflux pumps and increase the susceptibility of mycobacteria to antibiotics [20, 30]. Several efflux pump-encoding genes in *M*. *abscessus* were upregulated following exposure to frontline antibiotics [31, 32]. The effects of NMP and PLU on antibiotic tolerance and efflux pump activity in *M*. *abscessus* cells have not been reported. In this study, we investigated the effects of NMP and PLU on biofilm formation and antibiotic tolerance and of NMP on the utilization of exogenous fatty acids in the biosynthesis of cell surface and cellular lipids of *M*. *abscessus*. We observed that NMP and PLU inhibit *M*. *abscessus* biofilm formation at sub-MIC levels. We report that NMP severely inhibits the incorporation of radiolabeled ^14^C-palmitic acid into GPL, phosphatidylethanolamine (PE) and cardiolipin (CL) in *M*. *abscessus* biofilms. The biosynthesis of triacylglycerol (TAG) using ^14^C-palmitic acid and ^14^C-acetic acid was increased significantly in cellular lipids in log-phase and biofilm cells. NMP and PLU inhibited efflux pump activity in *M*. *abscessus* cells and respectively decreased the MICs of CLA and CIP against *M*. *abscessus* biofilms.

This is the first study on the metabolic utilization of exogenous long-chain fatty acids in the biosynthesis of polar cell surface and cellular lipids of log-phase and biofilm *M*. *abscessus* cells and the effect of NMP on such metabolic processes. This is the first report on the effects of NMP on TAG biosynthesis in *M*. *abscessus*. Our findings suggest that NMP causes a metabolic diversion of fatty acids from polar lipid biosynthesis to triacylglycerol synthesis in *M*. *abscessus*.

## 2. Materials and methods

### 2.1 Bacterial strain and culture conditions

*Mycobacterium abscessus* ATCC 19977 (rough morphotype), stored as a glycerol stock at -80°C, was inoculated into regular Middlebrook 7H9 medium (with 0.05% [w/v] Tween 80) + 10% [v/v] albumin dextrose catalase (ADC) and incubated with shaking at 37°C till the culture reached optical density at 600 nm of 0.6–0.8. In a modification of previous reports for growing mycobacteria in biofilms [33–36], we used an altered Middlebrook 7H9 medium and stationary growth conditions to culture *M*. *abscessus* cells as biofilms. The log-phase *M*. *abscessus* culture was diluted 1:100 in our biofilm-stimulating medium (BM; Middlebrook 7H9 broth lacking Tween 80 and supplemented with 2% [v/v] ADC and 0.5% [w/v] glucose) and incubated without shaking in multi-well plates at 37°C to promote biofilm formation.

### 2.2 Chemicals and reagents

The EPIs used in this study were 1-(1-naphthylmethyl)-piperazine (NMP; 97%; Thermo Fisher Scientific, Waltham, MA) and plumbagin (PLU; TCI America, Portland, OR; >98%). The following antibiotics were used in our assays: ciprofloxacin (CIP; 98%; Thermo Fisher Scientific), clarithromycin (CLA; >96%; Alfa Aesar, Haverhill, MA), amikacin (AMI; MP Biomedicals, Solon, OH) and cefoxitin (CEF; TCI America, >98%). Dimethyl sulfoxide (DMSO; MP Biomedicals), Tween 80, Resazurin sodium salt, 96-well plates were obtained from Thermo Fisher Scientific. Stock solutions of NMP (100 mg/mL or 10 mg/mL), PLU (2 mg/mL or 0.2 mg/mL), CIP (2 mg/mL or 0.2 mg/mL), CLA (2 mg/mL or 0.2 mg/mL), AMI (2 mg/mL or 0.2 mg/mL) and CEF (30 mg/mL or 3 mg/mL) were made in DMSO, filter-sterilized and stored as aliquots at -20°C. Difco Middlebrook 7H9 broth (Becton, Dickinson and Company, Sparks, MD), Albumin dextrose catalase (ADC; Becton, Dickinson and Company, Sparks, MD) and all other chemicals used were of highest purity and analytical grade. Mueller-Hinton broth (Oxoid/Thermo Fisher Scientific, UK) was supplemented with salts to provide 20–25 mg/L calcium and 10–12 mg/L magnesium.

### 2.3 Determination of minimum inhibitory and bactericidal concentrations of antimicrobials

*M*. *abscessus* was grown to mid log-phase in Middlebrook 7H9 containing Tween 80 and ADC as described above and diluted 1:100 into cation-adjusted Mueller-Hinton broth (CAMHB) containing 10% (v/v) ADC for determination of minimum inhibitory concentrations (MICs) and minimum bactericidal concentrations (MBCs) of antimicrobials used in this study. We followed modifications of procedures reported by others [37]. Assays were carried out in triplicate in 96-well plates (Thermo Fisher Scientific; Tissue Culture Treated; Cat. No. FB012931) containing the respective EPI or antibiotic in a volume of 200 μL and each well received 100 μL (~5 x 10^4^ colony forming units) of the culture. Control wells contained cells without antimicrobials or sterile medium only. The antibiotics CIP, CLA, AMI and CEF were tested at concentrations ranging from 0.25X to 40X of their respective MICs (CIP, 0.5 μg/mL; CLA, 1 μg/mL; AMI, 16 μg/mL; CEF, 16 μg/mL) reported for susceptible, rapidly growing, non-tuberculous mycobacteria [38]. The plates were then incubated at 37°C for 3 days. For determination of MICs, 25 μL of sterile 0.02% (w/v) resazurin (final concentration 88 μM) was added. The plates were incubated for another 24 h at 37°C. The resazurin (Alamar blue) assay is used for the quantitative colorimetric or fluorometric detection of cell viability or growth [39]. Viable cells metabolize resazurin (blue) to resorufin (pink) while non-viable cells remain blue. The lowest concentration that prevented a color change from blue was taken as the MIC for the respective antimicrobial. MICs were determined from three independent experiments (biological replicates) containing triplicate samples.

MBCs were determined by subculturing 20 μL of the cultures exposed to antimicrobials for 3-days into 180 μL of CAMHB containing 10% ADC in fresh 96-well plates followed by another 3-day incubation at 37°C. The resazurin assay was again performed as described above and the MBCs were determined. The lowest concentration that prevented a color change from blue was taken as the MBC for the respective antimicrobial. Three independent experiments (biological replicates) with samples in triplicate were performed to determine MBCs.

### 2.4 Assessment of biofilm formation, inhibition and eradication by antimicrobials

The formation of biofilms in our biofilm-stimulating medium, the effects of the antimicrobials on the inhibition of biofilm formation, and the ability of the antimicrobials to disperse established biofilms were assessed. We followed modifications of procedures reported by others [37, 40]. *M*. *abscessus* log-phase cultures were diluted 1:100 into biofilm-stimulating medium (BM) which was comprised of Middlebrook 7H9 broth lacking Tween 80 but containing 2% ADC and 0.5% glucose, (or, as control, Middlebrook 7H9 containing Tween 80 and ADC but without glucose) and 200 μL per well was dispensed into 96-well plates (Thermo Fisher Scientific; Tissue Culture Treated; Cat. No. FB012931) or 1 mL per well was dispensed into 12-well plates (Thermo Fisher Scientific; Tissue Culture Treated; Cat. No. FB012928). The antimicrobials (EPIs or antibiotics) were added at this time to determine their potential inhibitory effects on biofilm formation by log-phase, planktonic cells. Control wells did not receive any antimicrobials. The plates were placed into plastic bags which were sealed to minimize evaporation and incubated for 3 days at 37°C without any shaking. After 3 days, the medium and planktonic cells were removed, and the wells were gently rinsed in water to remove floating cells. Biofilm formation was measured using the crystal violet assay [35]. Biofilms in 96-well plates were stained with 150 μL of 1% (w/v) crystal violet and biofilms in 12-well plates were stained with 300 μL of 1% crystal violet for 10 min at room temperature. After three gentle washes with distilled water, the crystal violet was extracted from the biofilms by incubation for 10 min at room temperature with 150 μL of 95% ethanol (96-well plates) or 300 μL of 95% ethanol (12-well plates). The extracts (200 μL aliquots) were then transferred to fresh 96-well plates and the absorbance at 600 nm was recorded as a measure of biofilm formation using an accuSkan™ GO UV/Vis Microplate Spectrophotometer (Thermo Fisher Scientific, Waltham, MA). We followed previously established methods for *M*. *smegmatis* to determine the concentration of each antimicrobial that inhibited biofilm formation by 50% compared to controls not exposed to antimicrobials [40]. This was determined as the minimum biofilm inhibitory concentration (MBIC_50_). Three independent experiments (biological replicates) with triplicates in each experiment were performed to assess biofilm formation.

We followed previously reported procedures for *M*. *smegmatis*, with appropriate modifications, for determining the minimum biofilm eradication concentration (MBEC) for antimicrobials against *M*. *abscessus* in our assays [40]. Briefly, *M*. *abscessus* was allowed to form biofilms in the absence of antimicrobials. Then, the planktonic cells were removed and the antimicrobials were added and incubated further to determine their effects on dispersal of established biofilms. The effects of the antimicrobials used in our study on the dispersal of established biofilms were measured as follows: *M*. *abscessus* log-phase cultures were diluted 1:100 into biofilm-stimulating medium and 1 mL per well was aliquoted into 12-well plates. The plates were placed in plastic bags, sealed and incubated for 3 days at 37°C without shaking to allow biofilm formation in the absence of antimicrobials. After 3 days, the medium and planktonic cells were removed, and the wells were gently rinsed in water to remove floating cells. The antimicrobials were then added in 1 mL CAMHB + 10% ADC per well and the plates were incubated in sealed plastic bags for a further 3 days at 37°C without shaking. Control wells did not receive any antimicrobials. After the second 3-day incubation, the floating cells were removed, and the adhered biofilm layers were gently rinsed with water three times. Then, 300 μL of 1% (w/v) crystal violet was added per well and incubated for 10 min at room temperature. The stained biofilms were then rinsed gently with water three times and the crystal violet was eluted by incubation for 10 min at room temperature with 300 μL of 95% ethanol. The extracts (200 μL aliquots) were transferred to fresh 96-well plates and the absorbance at 600 nm was recorded as described above. Three independent experiments (biological replicates) were performed.

### 2.5 Checkerboard assays to evaluate the combinatory effects of antimicrobials against *M*. *abscessus*

Two-dimensional checkerboard assays were performed in 96-well plates to assess the combinatory effects of the antimicrobials used in our study following modifications of procedures reported by others [41, 42]. *M*. *abscessus* was grown to mid log-phase as described above, diluted 1:100 into CAMHB containing 10% ADC and 100 μL was placed in each well. Serial dilutions of the antimicrobial “A” (efflux pump inhibitor NMP or PLU) in CAMHB containing 10% ADC were placed in wells along the x-axis of the 96-well plates and serial dilutions of the antimicrobial “B” (antibiotic CIP, CLA, AMI or CEF) were placed in wells along the y-axis of the 96-well plates. Wells lacking either antimicrobial or lacking both antimicrobials served as controls. Total volume was 200 μL per well. The plates were placed in plastic bags which were sealed and incubated at 37°C for 3 days. For determination of MICs, after 3 days, 25 μL of sterile 0.02% (w/v) resazurin was added and the plates were incubated for another 24 h at 37°C. The lowest concentration that prevented a color change from blue was taken as the MIC for the respective antimicrobial. The MIC of each antimicrobial was determined at various concentrations of the other antimicrobial in the combination. *M*. *abscessus* exhibited complete viability in our assays at concentrations ≤ 0.5X MIC of each antimicrobial in our study. Therefore, the combinatory MIC and fractional inhibitory concentration (FIC) values and Modulatory Factors were determined as follows:

MIC_AB_ = MIC_A_ in the presence of B at ≤ 0.5X MIC_B_

MIC_BA_ = MIC_B_ in the presence of A at ≤ 0.5X MIC_A_

FIC_A_ = MIC_AB_/ MIC_A_

FIC_B_ = MIC_BA_/ MIC_B_

Fractional Inhibitory Concentration Index (FICI) = FIC_A_ + FIC_B_

Modulatory Factor = MIC of antibiotic/ MIC of antibiotic + EPI

The FICI values for each combination were determined from three independent experiments (biological replicates). The lowest values (FICI_L_) and the highest values (FICI_H_) observed in our assays were interpreted as follows: FICI ≤ 0.5 = “synergy”; FICI > 0.5–4.0 = “no interaction”; FICI > 4.0 = “antagonism”, following established criteria for interpretation of FICI values and used in studies on mycobacteria [42, 43].

### 2.6 Metabolic radiolabeling of log-phase and biofilm cells of *M*. *abscessus* with ^14^C-palmitate and ^14^C-acetate

We followed a modification of our previously published protocol for metabolically radiolabeling mycobacterial cells [11]. All radiolabeling experiments were performed three times independently (three biological replicates) with duplicate samples in each experiment. Briefly, the planktonic, log-phase (0 day) *M*. *abscessus* cells at OD_600_ ~ 0.8 were exposed to 600 μg/mL NMP or DMSO for 30 min prior to radiolabel incorporation. *M*. *abscessus* cells in biofilms were obtained by dilution of the log-phase culture 1:100 into biofilm medium containing 600 μg/mL NMP or DMSO. This diluted culture was placed in 24-well plates (3 mL per well) which were sealed with parafilm to prevent evaporation and incubated for biofilm formation for 3 days. Biofilm cells (floating and submerged) were collected from the 24-well plate at day 3 and resuspended by vortexing prior to radiolabeling. Metabolic radiolabeling was performed with [1-^14^C]palmitic acid (56.67 mCi/mmol; Perkin Elmer Health Sciences Inc., Shelton, CT), 1 μCi/ 3 ml cells) for 6 h or [1-^14^C]acetic acid (56.6 mCi/mmol; American Radiolabeled Chemicals, St. Louis, MO, 2 μCi/ 3 ml of cells) for 24 h with shaking at 37°C. Following radiolabeling, the cells were pelleted by centrifugation at 5000 x g, 10 min and the cell pellet was used for extraction of lipids as described below.

### 2.7 Extraction and analysis of radiolabeled lipids

Mycobacterial cell surface and cellular lipids were extracted by following procedures established by others previously [44, 45], with slight modifications. Briefly, *M*. *abscessus* cell pellets collected after radiolabeling, were vortexed for 1 min with 0.3 g of glass beads (4 mm diameter) and re-vortexed after addition of 0.5 mL water. After centrifugation at 5000 x g, 10 min the aqueous supernatant was acidified and extracted by vortexing for 1 min with 8 mL of chloroform:methanol (2:1, v/v). Following phase separation after addition of 0.5 mL of water and vortexing, cell surface lipids collected in the lower organic phase were transferred to fresh tubes. The aqueous phase was extracted again using 5 mL of chloroform with vortexing and the extracts were pooled as “cell surface lipids”. The cell pellet collected after extraction of surface lipids was resuspended in 6 mL of chloroform:methanol (2:1, v/v) and “cellular lipids” were extracted with gentle shaking at room temperature overnight followed by addition of 0.5 mL acidified water, vortexing and collection of lower organic phase. Equal amounts of total radioactivity (approximately 50,000 dpm per sample) were analyzed by silica-thin layer chromatography (TLC; Merck silica gel 60, MilliporeSigma, Burlington, MA). The polar lipids were resolved using chloroform:methanol:water (65:25:4, by volume) as the solvent system to identify the resolved lipids and the TLC plates were exposed to autoradiographic film. Radioactive bands corresponding to glycopeptidolipids (GPL), phosphatidylethanolamine (PE), cardiolipin (CL) and phosphatidylinositol/ phosphatidylinositol mannosides (PI/PIMs) were identified based on their R_f_ values reported in this solvent system by others previously [46]. Standard PE and CL (850705P, 710335C Avanti Polar Lipids, Alabaster, AL) were also used to ascertain the relative mobilities of PE and CL in this TLC solvent system. Metabolic radiolabeling of the neutral storage lipid TAG in the cellular lipids of log-phase and 3-day biofilm cells of *M*. *abscessus* was analyzed by silica-TLC using hexane:diethyl ether:acetic acid (80:20:2, v/v/v). Standard TAG and fatty acids (FA) were used to verify the relative mobilities of TAG and FA in this solvent system. The respective lipid bands were scraped from TLC plates and radioactivity was quantified by liquid scintillation counting. Three independent experiments were performed with duplicates in each experiment.

### 2.8 Efflux pump activity assays

The effects of the EPIs on ethidium bromide (EtBr) accumulation and efflux were measured for assessing efflux pump activity using methods modified from a previous report [30]. Active efflux pumps in the bacterial cell would cause a rapid loss of EtBr fluorescence while any EPI that blocks such efflux pumps would inhibit such a decrease in fluorescence. Log-phase cells were used since they are not clumped together as biofilm cells, thereby allowing better measurements of the EtBr transport kinetics. The planktonic *M*. *abscessus* cells in log-phase growth were collected by centrifugation, the supernatant was discarded, and the pellet was resuspended and washed in sterile phosphate-buffered saline (PBS) and kept at 37°C. The OD_600_ was adjusted to 0.4 and 100 μL of the bacterial suspension was used in the EtBr accumulation assays using EtBr concentrations from 0.125 μg/mL to 4 μg/mL. EtBr accumulation was measured by reading the fluorescence data every 1 min using an Agilent BioTek Synergy LX fluorescence plate reader (Agilent Technologies, Santa Clara, CA) by measuring fluorescence emission at 590 nm after excitation at 530 nm (Red filter). EtBr concentration of 0.25 μg/mL was used to assess the effect of temperature on EtBr accumulation at 25°C and 37°C.

For the efflux assays, the conditions used were a modification of a previously reported procedure [30]. *M*. *abscessus* cells in log-phase growth were first allowed to accumulate EtBr at 25°C without glucose in the presence of verapamil at 0.5X MIC to block the initial efflux of EtBr prior to the assay for 60 min with an EtBr concentration that led to maximum accumulation (4 μg/mL, equivalent to 0.03X MIC of EtBr). The EtBr-loaded cells were collected by centrifugation and washed in ice-cold PBS. EtBr efflux was examined in the presence or absence of glucose (2%, w/v). Earlier studies done by others examined the modulatory effects of EPIs at 0.5X MIC levels [47]. The EPIs NMP and PLU were added to the planktonic, log-phase cells in PBS at 600 μg/mL and 5 μg/mL respectively for the 60 min assay period. Verapamil (VER; 512 μg/mL; 0.5X MIC) was used as a positive control. The EtBr efflux from the *M*. *abscessus* cells was measured by reading the fluorescence data as described above. The accumulation and efflux graphs were obtained by normalizing the fluorescence at respective time-point with the fluorescence at the initial time-point. The efflux activity as a percentage of the control at 60 min was calculated by dividing the fluorescence value at 60 min by the initial time-point (0 min) for respective samples. The values obtained were subtracted from 1. The values for the EPI-treated samples were divided by the values for the control samples and multiplied by 100 to obtain “Efflux Activity (% of control at 60 min)”.

### 2.9 Statistical analyses

Unless otherwise mentioned, the experiments in this study were repeated at least three independent times (biological replicates) with duplicates or triplicates in individual experiments. In the assessment of biofilm formation by CV assays, EtBr accumulation and efflux assays, One-Way ANOVA and post-hoc Tukey test was performed with Sigma Plot 14.5 to determine significance. The experiments involving radioactive incorporation and lipid analyses were repeated three independent times (three biological replicates) with duplicates in each experiment. A representative experiment, with the values as average ± standard deviation from duplicate measurements, is depicted graphically. Student’s t-Test (two-tailed; unpaired) was performed to determine significance of the observed results.

## 3. Results

### 3.1 *Mycobacterium abscessus* biofilms show greater tolerance of efflux pump inhibitors

**Fig 1:**
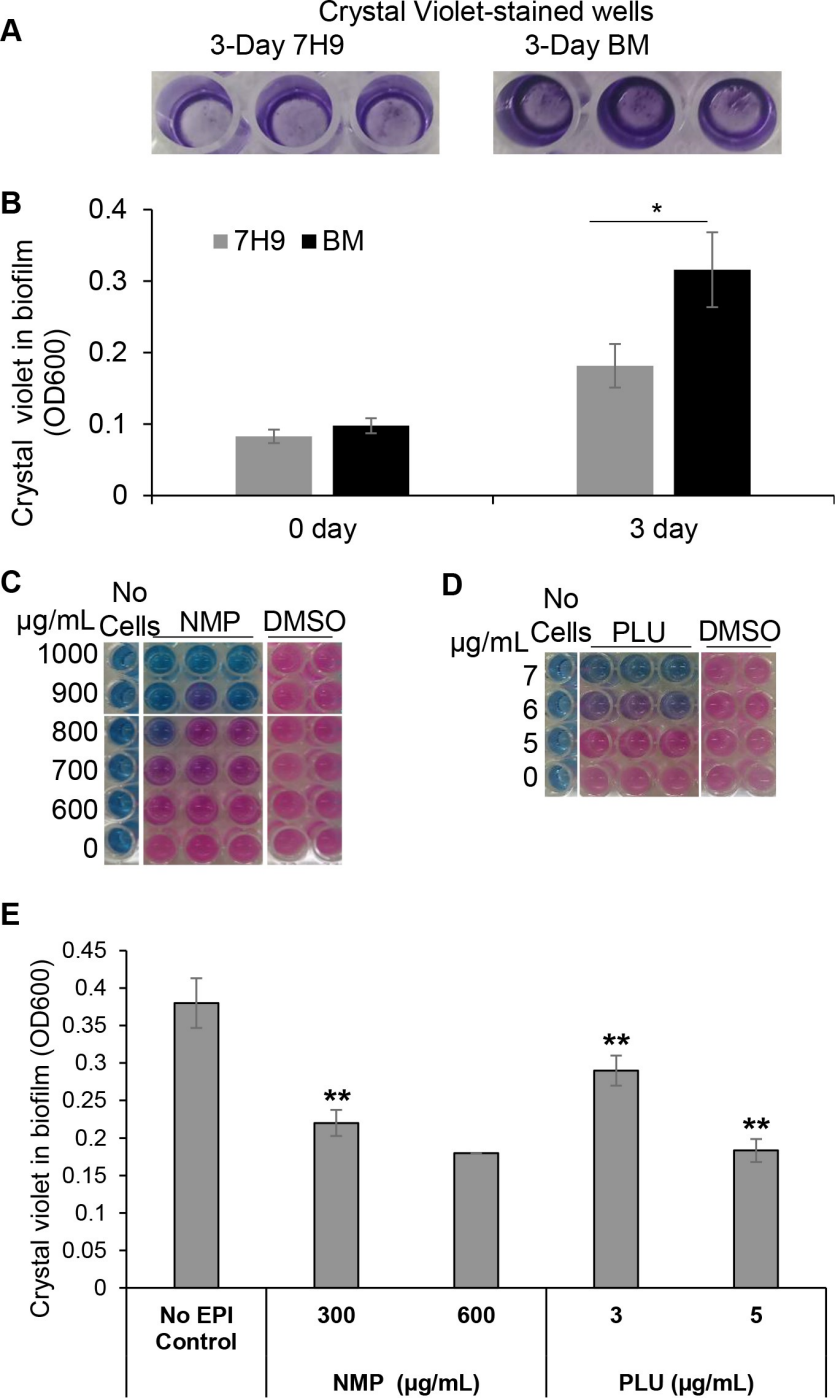
Efflux pump inhibitors NMP and PLU inhibit the viability of *Mycobacterium abscessus* biofilms. **A**, *M*. *abscessus* cells grown under stationary conditions in Middlebrook 7H9 medium (7H9) and biofilm-promoting medium (BM) stained with crystal violet at day 3. **B**, Quantification of crystal violet staining by *M*. *abscessus* biofilms in 7H9 and BM. Three independent experiments were performed. Values are expressed as average ± standard deviation from triplicates in a representative experiment. *, p<0.05; 7H9 vs. BM at day 3. One-Way ANOVA and post-hoc Tukey test was performed with Sigma Plot 14.5 to determine significance. **C**, **D** MICs of efflux pump inhibitors 1-(1-naphthylmethyl)-piperazine (NMP) and plumbagin (PLU) against *M*. *abscessus* biofilms. Cells were exposed to efflux pump inhibitors for 3 days in BM and then stained with resazurin for 24 h. Pink color shows the presence of viable cells and blue color indicates lack of viable cells. **E,** NMP and PLU inhibit biofilm formation at sub-MIC levels by *M*. *abscessus* in BM. **, p<0.01; Vs. No EPI control.

We determined the MICs and MBCs of the antimicrobials used in this study against *M*. *abscessus* in planktonic, log-phase growth condition in cation-adjusted Mueller-Hinton broth using the resazurin microbroth dilution assay. The lowest concentration that prevented a color change from blue to pink, indicating complete loss of cell viability and growth, was determined as the MIC (Table 1). The Middlebrook 7H9 medium used for culturing mycobacteria contains the detergent Tween 80 which is known to remove mycobacterial cell surface-exposed lipids [48]. This removal of cell surface molecules could affect biofilm formation. Therefore, we cultured *M*. *abscessus* in our biofilm-stimulating medium that does not contain Tween 80 and which is supplemented with glucose that is known to enhance mycobacterial biofilm formation [33–36, 49]. The *M*. *abscessus* cells were incubated under stationary conditions to allow biofilm formation. Biofilm formation was measured after 3 days by staining with crystal violet. Our findings showed that, under stationary conditions, biofilm formation by *M*. *abscessus* was about 2-fold higher in our novel biofilm stimulating medium than in the regular 7H9 medium after three days (Fig 1A and 1B). The MICs of the EPIs NMP and PLU against *M*. *abscessus* biofilms have not been determined previously. Therefore, we exposed *M*. *abscessus* biofilms to these EPIs and determined their MICs using the resazurin assay. The MICs for NMP and PLU were higher in *M*. *abscessus* biofilms grown in biofilm-stimulating medium than in planktonic cells grown in the Mueller-Hinton broth. In our biofilm-stimulating medium, the MICs for NMP and PLU were 1000 μg/mL and 7 μg/mL, respectively (Fig 1C and 1D). These MIC values were higher when compared to planktonic *M*. *abscessus* in CAMHB medium (Table 1).

**Table 1:** Effects of antimicrobials on viability and biofilm-formation by *M*. *abscessus*.

Antimicrobial	MIC^a^ (μg/mL)	MBC^a^ (μg/mL)	MBIC_50_^b^ (μg/mL)
Efflux Pump Inhibitor			
NMP	600	800	600
PLU	7	10	5
Antibiotic			
CIP	10	20	8
CLA	10	14	5
AMI	12	24	8
CEF	200	400	150

MIC = Minimum Inhibitory Concentration; MBC = Minimum Bactericidal Concentration; MBIC_50_ = Minimum Biofilm Inhibitory Concentration that inhibited biofilm formation by 50% compared to controls. ^a^MIC and MBC were determined using log-phase, planktonic *M*. *abscessus* in CAMHB. ^b^MBIC_50_ was determined using log-phase, planktonic *M*. *abscessus* induced for biofilm formation in BM.

### 3.2 EPIs and antibiotics inhibit *M*. *abscessus* biofilm formation at sub-MIC levels

NMP was recently shown to inhibit biofilm formation in *M*. *tuberculosis* and the biofilm-blocking effect of NMP was linked with its inhibition of ABC transporter gene expression [24]. PLU was reported to be a competitive inhibitor of an ABC transporter [21, 25, 50]. We examined the effects of NMP and PLU on biofilm formation by log-phase cells of *M*. *abscessus* using the crystal violet assay. At sub-MIC levels, both the EPIs, NMP and PLU, inhibited biofilm formation by about 50% (Fig 1E). We also determined the concentrations of the antibiotics that inhibited biofilm formation by 50% when compared to the controls (Table 1). We observed that the MBIC_50_ values were slightly lower than MIC and MBC values suggesting that sub-MIC levels of all antimicrobials in our study (except NMP) could inhibit biofilm formation by log-phase *M*. *abscessus* (Table 1). Furthermore, we examined whether the antimicrobials used in this study could disperse established biofilms. We found that none of the antimicrobials used in this study could eradicate pre-formed biofilms in a dose-dependent manner.

### 3.3 NMP increases the effectiveness of major antibiotics against *Mycobacterium abscessus*

We investigated the effects of the EPIs, NMP and PLU, on the sensitivity of *M*. *abscessus* to major antibiotics like CIP, CLA, AMI and CEF. The effects of NMP and PLU on the MICs of these antibiotics against *M*. *abscessus* have not been reported. We performed checkerboard assays to determine the effects of the EPIs in combination with the antibiotics. The FICI values showed that the EPIs and antibiotics exhibited “no interaction” in their combinatory action on *M*. *abscessus* (Table 2). However, both NMP and PLU decreased the MICs of CIP and CLA by 5-fold (Table 3). The MIC of AMI was decreased 3-fold by both EPIs.

**Table 2:** Combinatory effects of EPIs and antibiotics against *M*. *abscessus*.

Combination (EPI + Antibiotic)	FICI_L_^a^	FICI_H_^b^	Combinatory Effect
NMP + CIP	0.53	0.87	No interaction
NMP + CLA	0.53	0.90	No interaction
NMP + AMI	0.67	1.00	No interaction
NMP + CEF	0.77	1.47	No interaction
PLU + CIP	0.88	0.93	No interaction
PLU + CLA	0.68	0.91	No interaction
PLU + AMI	0.76	1.05	No interaction
PLU + CEF	0.9	1.41	No interaction

^a^FICI_L_ = the lowest FICI observed in this assay

^b^FICI_H_ = the highest FICI observed in this assay. Checkerboard assays were performed with log-phase, planktonic *M*. *abscessus* in CAMHB.

**Table 3:** Effect of EPIs on the MICs of antibiotics against *M*. *abscessus*.

	MIC (μg/mL)							
	NMP (μg/mL)				PLU (μg/mL)			
	0	200	400	MF_H_	0	3	5	MF_H_
CIP	10	2	2	5	10	10	2	5
CLA	10	2	2	5	10	4	2	5
AMI	12	4	4	3	12	8	4	3
CEF	200	140	100	2	200	140	140	1.4

Modulatory Factor—MF_H_ = MIC of antibiotic alone/ MIC of antibiotic + EPI at the higher dose. MIC assays were performed with log-phase, planktonic *M*. *abscessus* in CAMHB.

### 3.4 Metabolic utilization of radiolabeled fatty acids for lipid biosynthesis in *M*. *abscessus* is inhibited by NMP

*M*. *abscessus* in phagolysosomes could utilize fatty acids from lipid droplets in the macrophage as well as the lipid-rich environment of the granuloma for bacterial lipid biosynthesis like *M*. *tuberculosis* [3–5]. However, the biosynthesis of lipids using fatty acids imported into *M*. *abscessus* from exogenous sources have not been examined before. Since NMP showed an inhibitory effect on biofilm formation in our assays above, we investigated the effects of NMP on the utilization of exogenous long-chain fatty acids in the biosynthesis of cell surface or cellular lipids in *M*. *abscessus* biofilms and compared them with *M*. *abscessus* in log-phase growth. We used ^14^C-labeled palmitic acid (C16:0) to metabolically radiolabel the polar lipids synthesized by *M*. *abscessus* during planktonic, log-phase growth and during biofilm formation. We investigated the effects of sub-MIC levels of NMP on such lipid biosynthesis. The cell surface and cellular lipids of *M*. *abscessus* were isolated from 3-day biofilms and compared with radiolabeled lipids isolated from log-phase *M*. *abscessus*.

**Fig 2:**
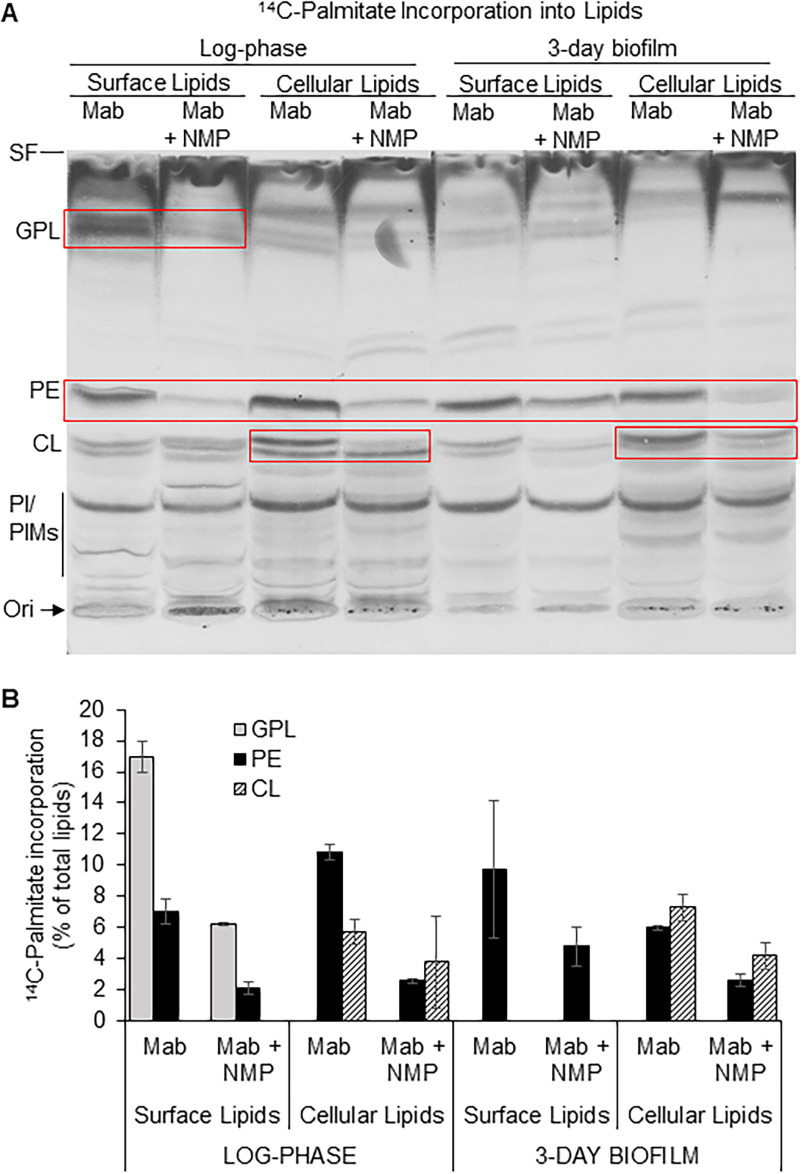
NMP inhibits the incorporation of ^14^C-palmitic acid into glycopeptidolipids, phosphatidylethanolamine and cardiolipin in *M*. *abscessus*. Cell surface and cellular lipids from log-phase and 3-day biofilm cells of *M*. *abscessus* metabolically radiolabeled with ^14^C-palmitate for 6 h in the presence or absence of NMP were analyzed by silica-TLC using chloroform:methanol:water (65:25:4, v/v/v) as solvent system. **A,** Autoradiogram of TLC plate from a representative experiment shown. Equal amounts of total lipid radioactivity loaded in each lane. Three independent experiments performed. Red boxes indicate lipids that showed significant changes upon NMP treatment across experiments. SF, solvent front; GPL, glycopeptidolipids (R_f_ ~0.84); PE, phosphatidylethanolamine (R_f_ ~0.48); CL, cardiolipin (R_f_ ~0.36); PI, phosphatidylinositol; PIMs, phosphatidylinositol mannosides (R_f_ ~0.26–0.1); Ori, origin/ sample loading zone. **B**, Normalized quantification of lipids indicated in red boxes in panel A. Radioactivity incorporated into each lipid is shown as a percent of radioactivity in respective total lipid extract. Average ± SD of duplicates from a representative experiment is shown. Student’s *t*-Test was performed to determine significance (*M*. *abscessus* vs. *M*. *abscessus* + NMP) *, p *<* 0.05. Mab, *Mycobacterium abscessus*.

We observed that metabolic incorporation of radiolabeled palmitic acid in the surface lipids of log-phase cells was predominantly (17 ± 1% of total lipids) into GPL and that this incorporation was inhibited by NMP to about 6 ± 0.1% of total lipids (Fig 2A and 2B). Thus, NMP inhibited incorporation of radiolabel into GPL by nearly 65% in log-phase *M*. *abscessus*. Unlike log-phase cells, GPL was not the predominantly radiolabeled lipid in biofilms, and it accounted for only about 5 ± 3% of cell surface lipids. In cellular lipids of both log-phase and biofilm *M*. *abscessus*, PE, CL and PI/PIMs were the predominantly radiolabeled lipids. Radiolabel in PE was about 7 ± 0.8% of total cell surface lipids and 11 ± 0.5% of total cellular lipids in log-phase *M*. *abscessus*. In untreated biofilm cells, PE accounted for about 6 ± 0.2% of radioactivity in total cellular lipids. CL accounted for approximately 6 ± 0.8% of total cellular lipid radioactivity in log-phase *M*. *abscessus*. In untreated biofilm cells, radioactivity incorporated into CL was about 7 ± 0.9% of total cellular lipid radioactivity. NMP significantly inhibited the incorporation of the exogenously-provided radiolabeled long-chain fatty acid into PE. Incorporation of radiolabel into PE was decreased by approximately 70% in surface lipids and by nearly 75% in cellular lipids in log-phase *M*. *abscessus*. In *M*. *abscessus* biofilms, incorporation of radiolabel into PE in cellular lipids was decreased by approximately 50%. The biosynthesis of CL from exogenous fatty acids in cellular lipids of *M*. *abscessus* biofilms was significantly inhibited by NMP by nearly 40% compared to untreated controls. NMP did not significantly affect incorporation of exogenous long-chain fatty acids into PI/PIMs (Fig 2A and 2B).

### 3.5 NMP affects the metabolic incorporation of ^14^C-acetate into PE and CL in the cellular lipids of log-phase *M*. *abscessus*

**Fig 3:**
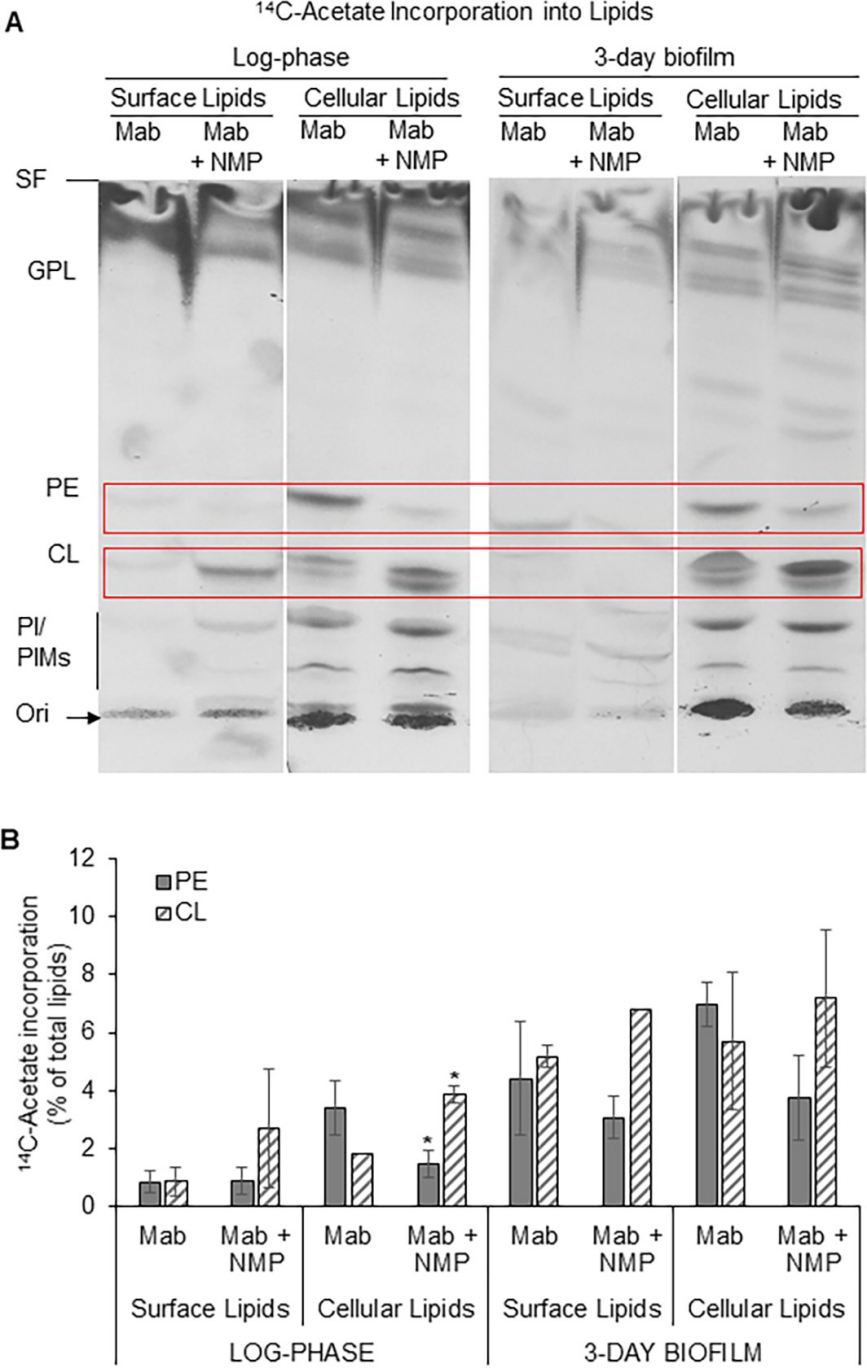
Metabolic incorporation of ^14^C-acetate into PE and CL in cellular lipids of log-phase *M*. *abscessus* is affected by NMP. **A,** Autoradiogram of TLC plate from a representative experiment shown. Equal amounts of total lipid radioactivity loaded in each lane. Three independent experiments performed. Cell surface and cellular lipids of log-phase and biofilm cells of *M*. *abscessus* were extracted and analyzed by silica-TLC using chloroform:methanol:water (65:25:4, v/v/v) as solvent system. SF, solvent front; GPL, glycopeptidolipids; PE, phosphatidylethanolamine; CL, cardiolipin; PI, phosphatidylinositol; PIMs, phosphatidylinositol mannosides; Ori, origin/ sample loading zone. **B**, Normalized quantification of lipids indicated in red boxes in panel A. Radioactivity incorporated into each lipid is shown as a percent of radioactivity in respective total lipid extract. Average ± SD of duplicates from a representative experiment is shown. Student’s *t*-Test was performed to determine significance (*M*. *abscessus* vs. *M*. *abscessus* + NMP). *, p *<* 0.05. Mab, *Mycobacterium abscessus*.

We examined the metabolic incorporation of ^14^C-acetate into lipids of *M*. *abscessus* in log-phase and in biofilms. Since acetate is the precursor of the fatty acid synthase that provides endogenously-synthesized fatty acids for lipid biosynthesis, radiolabeling studies utilizing ^14^C-acetate could reveal the effects of NMP on such lipid biosynthetic pathways in *M*. *abscessus*. We observed that NMP inhibited the incorporation of ^14^C-acetate into PE and increased the radiolabel incorporation into CL in the cellular lipids of log-phase *M*. *abscessus* (Fig 3A). The radioactivity in PE decreased from about 3 ± 0.9% of total lipids in untreated cells to about 1.5 ± 0.5% in NMP-treated cells. Radioactivity in CL increased 2-fold in the presence of NMP (Fig 3B).

### 3.6 Metabolic utilization of ^14^C-palmitate and ^14^C-acetate for triacylglycerol biosynthesis in *M*. *abscessus* is increased by NMP

**Fig 4:**
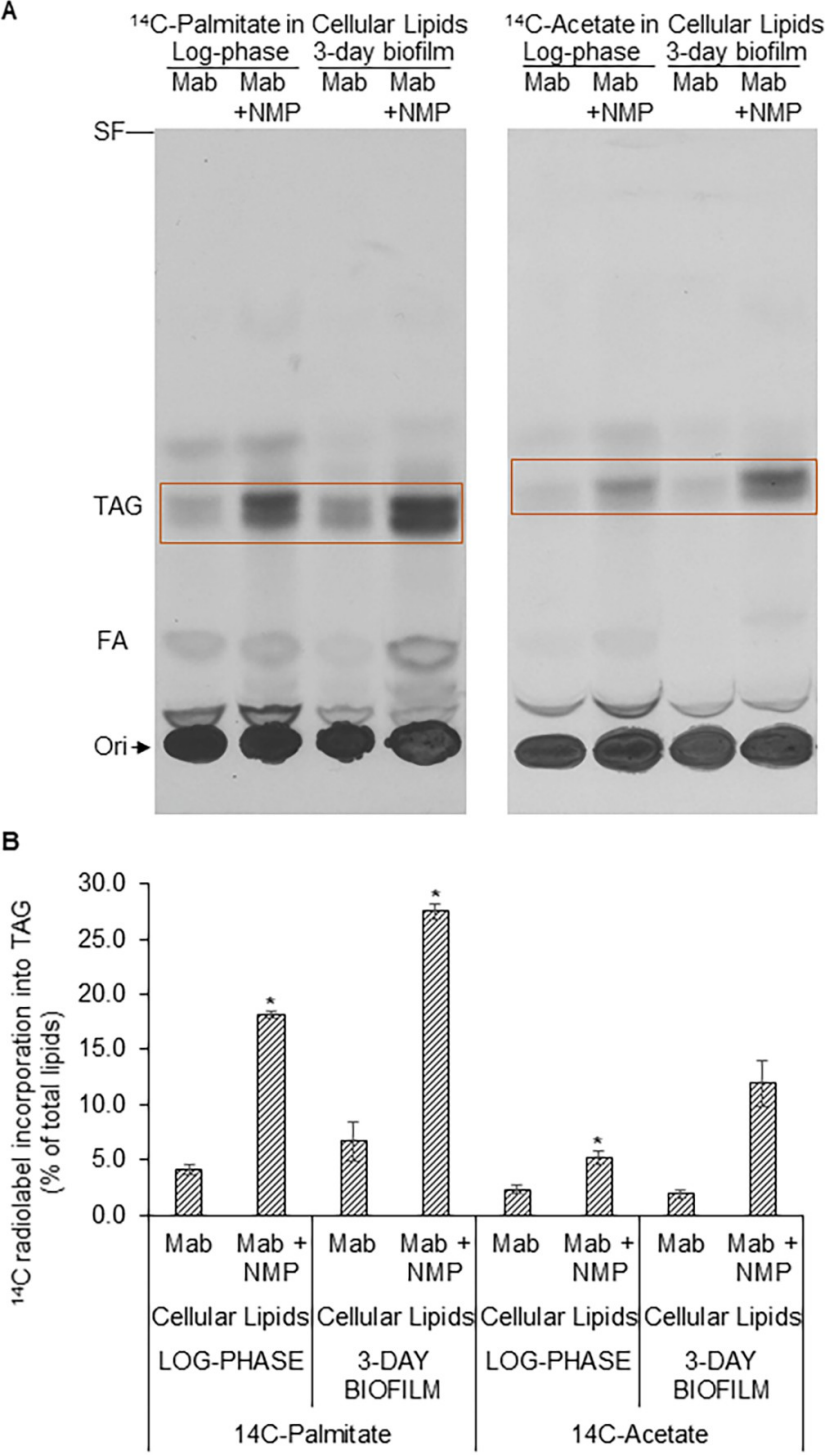
Incorporation of ^14^C-palmitate and ^14^C-acetate into triacylglycerol in *M*. *abscessus* is increased by NMP. Metabolic radiolabeling of the neutral storage lipid triacylglycerol (TAG) in the cellular lipids of log-phase and 3-day biofilm cells of *M*. *abscessus* with ^14^C-palmitate for 6 h or ^14^C-acetate for 24 h in the presence or absence of NMP was analyzed by silica-TLC using hexane:diethylether:acetic acid (80:20:2, v/v/v). **A,** Autoradiogram of TLC plate from a representative experiment shown. Equal amounts of total lipid radioactivity loaded in each lane. Three independent experiments performed. Orange boxes indicate TAG that showed significant changes upon NMP treatment across experiments. SF, solvent front; TAG, triacylglycerol (R_f_ ~0.36); FA, fatty acid (R_f_ ~0.13); Ori, origin/ sample loading zone. **B**, Normalized quantification of TAG indicated in orange boxes in panel A. Radioactivity incorporated into TAG is shown as a percent of radioactivity in respective total lipid extract. Average ± SD of duplicates from a representative experiment is shown. Student’s *t*-Test was performed to determine significance (*M*. *abscessus* vs. *M*. *abscessus* + NMP) *, p *<* 0.05. Mab, *Mycobacterium abscessus*.

The effects of chemosensitizers or EPIs such as NMP on TAG biosynthesis from exogenous fatty acids in *M*. *abscessus* have not been investigated. We observed that NMP increased the metabolic incorporation of ^14^C-palmitate into the neutral storage lipid TAG in the cellular lipids of log-phase and biofilm *M*. *abscessus* cells (Fig 4A; left panel). Incorporation of ^14^C-palmitate into TAG in log-phase cells was increased by NMP from about 4 ± 0.5% of total lipids to about 18 ± 0.3% of total lipids. In biofilm cellular lipids, incorporation of ^14^C-palmitate increased from about 7 ± 1.7% of total lipids in the absence of NMP to about 28 ± 0.7% in the presence of NMP (Fig 4B). The incorporation of ^14^C-acetate into TAG in cellular lipids of log-phase *M*. *abscessus* was increased from 2 ± 0.4% in the control cells not exposed to NMP to 5 ± 0.6% in the presence of NMP. In biofilm cells, incorporation of this radiolabel increased from about 2 ± 0.3% to 12 ± 2% (Fig 4A; right panel & Fig 4B).

### 3.7 NMP and PLU decrease efflux pump activity in *Mycobacterium abscessus*

**Fig 5:**
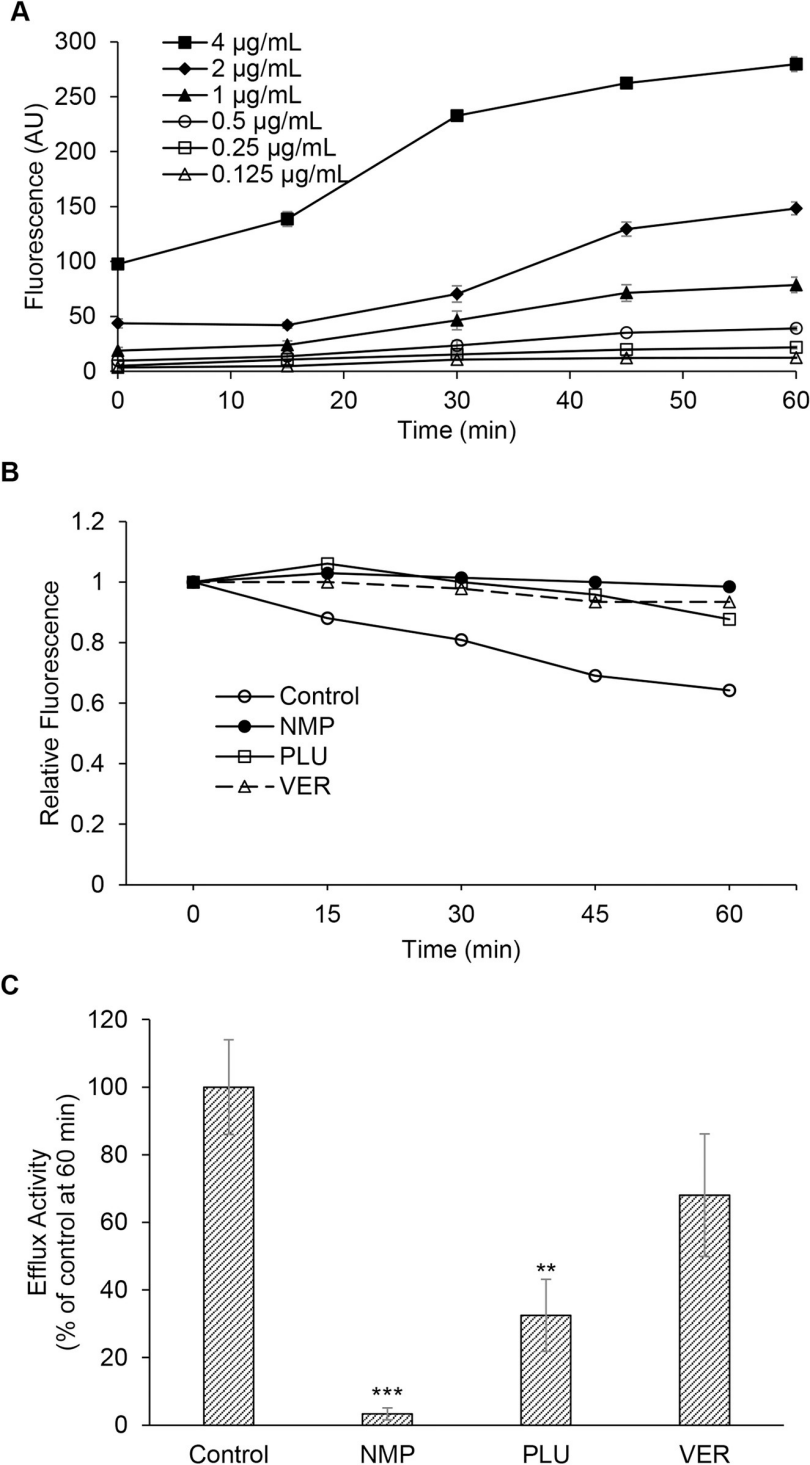
NMP and PLU inhibit efflux activity in *Mycobacterium abscessus*. **A,** EtBr accumulation in *Mycobacterium abscessus* is concentration-dependent. Intracellular accumulation of EtBr at concentrations from 0.125 μg/mL to 4 μg/mL was measured. Fluorescence level (arbitrary units) measurements were obtained every minute for 60 minutes. Three independent experiments performed. Values are expressed as average ± standard deviation from triplicates in a representative experiment. **B,** EtBr efflux inhibition by NMP and PLU. Intracellular EtBr fluorescence of *M*. *abscessus* cells at 25°C treated with the efflux pump inhibitors NMP, PLU were compared to DMSO control and verapamil served as reference EPI. The cells were preloaded with EtBr at 4 μg/mL in the presence of verapamil at 0.5X MIC to serve as initial blocker. NMP and PLU were dissolved in DMSO. Three independent experiments performed. Values are expressed as average from triplicates in a representative experiment. **C**, Quantification of efflux activity after 60 min of *M*. *abscessus* cells at 25°C treated with the efflux pump inhibitors NMP and PLU compared to solvent control. Three independent experiments performed. Values are expressed as average ± standard deviation from triplicates in a representative experiment. One-way ANOVA and post-hoc Tukey test was performed with Sigma Plot 14.5 to determine significance difference between the EPI treated cells and its control. **, p<0.01; ***, p<0.001.

The effects of NMP and PLU on efflux pump activity in *M*. *abscessus* have not been investigated. The efflux of EtBr is well-established as an indicator of efflux pump activity [30]. Therefore, we investigated the EtBr transport activity of *M*. *abscessus* in planktonic, log-phase growth conditions. We observed that the accumulation of EtBr by *M*. *abscessus* was dependent on the concentration of EtBr in the assay and selected 4 μg/mL EtBr for the efflux assays (Fig 5A). We found that *M*. *abscessus* exposed to sub-MIC levels of the EPIs NMP (600 μg/mL) and PLU (5 μg/mL) retained EtBr fluorescence intracellularly at higher levels compared to the controls suggesting the blocking of efflux activity by these EPIs (Fig 5B). NMP inhibited efflux activity by nearly 97% and PLU inhibited efflux activities in *M*. *abscessus* cells by approximately 70% compared to the control at 60 min (Fig 5C). VER which was used as a positive control inhibited efflux by nearly 30% under our assay conditions. The accumulation of EtBr by *M*. *abscessus* was not significantly affected by incubation temperature at 60 min (S1 Fig). We tested the effects of the EPIs on EtBr efflux in the presence and absence of glucose, which serves to energize certain classes of efflux pump proteins. We observed that glucose did not significantly affect the efflux activities or the inhibitory effects of the EPIs in planktonic, log-phase *M*. *abscessus* (S2 Fig).

## 4. Discussion

*M*. *abscessus* is a biofilm-forming bacterium that causes surgical site infections and severe pathogenesis in cystic fibrosis patients [1, 26, 27, 51]. The Tween 80 detergent in Middlebrook 7H9 medium causes solubilization of cell surface molecules potentially involved in biofilm formation and are generally avoided when studying biofilms [35, 48, 52]. Glucose contributes to the formation of extracellular polymeric substances involved in biofilm formation [33, 36, 49]. We observed that, under stationary conditions, biofilm formation by *M*. *abscessus* was about 2-fold higher on day 3 in our biofilm-stimulating medium lacking Tween 80 and supplemented with 2% ADC and 0.5% glucose when compared to *M*. *abscessus* in the regular Middlebrook 7H9 medium (Fig 1A and 1B).

We utilized the resazurin (Alamar Blue) microplate assay for determining the viability states of *M*. *abscessus* in our assays. The resazurin assay is well-established as a colorimetric or fluorometric assay for cell viability and growth after exposure to antimycobacterials [38, 39]. Under stationary conditions, *M*. *abscessus* cells in the biofilm-stimulating medium developed higher tolerance to the EPIs compared to *M*. *abscessus* in the regular 7H9 medium potentially due to the upregulation of efflux pump genes in the biofilms [28].

EPIs have been investigated for their biofilm inhibitory properties in several Gram-negative bacteria and NMP has been shown to inhibit biofilm formation in such bacteria [16, 22]. NMP and PLU have been reported to inhibit efflux pumps belonging to the RND and ABC families in other bacteria and RND family efflux pumps have been shown to be involved in the export of lipids to the mycobacterial cell surface [16, 17, 20]. Our findings which show that NMP inhibits *M*. *abscessus* biofilm formation (Table 1 and Fig 1E) are in agreement with similar biofilm-inhibitory effects on *M*. *tuberculosis* [24]. These observations could be attributed to its effects on *M*. *abscessus* efflux pumps belonging the ABC or RND families that are known to export lipids to the cell surface [17]. Our results suggest that sub-MIC levels of all antimicrobials in our study (except NMP) could inhibit biofilm formation by log-phase *M*. *abscessus* (Table 1).

Efflux pumps have been reported to pump out the antibiotics CIP, CLA, AMI and CEF, which belong to the fluoroquinolone, macrolide, aminoglycoside and cephalosporin (β-lactam) classes respectively and are effective against infections by the *M*. *abscessus* complex [2, 29]. NMP and PLU have been reported to be good inhibitors of efflux pumps in other bacteria [20, 25, 53]. We conclude that our FICI values indicate “No interaction” (Table 2) following the established criteria for interpreting FICI in all studies including those in mycobacteria [42, 43]. We observed 5-fold reductions in the MICs of CIP and CLA against *M*. *abscessus* in the presence of sub-MIC levels of NMP (Table 3). NMP is known to inhibit RND efflux pumps involved in the efflux of macrolides, β-lactams and fluoroquinolones and PLU was reported to be a competitive inhibitor of an ABC transporter [21, 25, 50]. PLU has been reported to block the ABC efflux pump that pumps out β-lactam, aminoglycoside and macrolide antibiotics [25, 29]. PLU caused a 3-fold reduction in the MIC of AMI (Table 3). Our findings taken together with these previous reports suggest that NMP might be inhibiting RND efflux pumps involved in the efflux of CIP and CLA in *M*. *abscessus*. Our findings suggest that PLU might be inhibiting the activity of ABC efflux pumps involved in the efflux of AMI in *M*. *abscessus*. Further studies are needed to investigate these observations.

Our study provides novel findings on the metabolic incorporation of radiolabeled long-chain fatty acids from an exogenous source into *M*. *abscessus* cell surface and cellular lipids during log-phase and biofilm growth conditions. Such studies have not been reported previously. We show for the first time that NMP inhibits the utilization of ^14^C-palmitate in the biosynthesis of GPL, PE and CL in *M*. *abscessus* cells (Fig 2A and 2B). Our findings that show the inhibitory effects of NMP on cellular polar lipid biosynthesis reveal a novel metabolic effect of NMP which could be attributed to its chemosensitizing property that causes membrane destabilization at sub-MIC levels reported earlier by others [23]. The biosynthesis of polar lipids such as GPLs in non-tuberculous mycobacteria have been extensively investigated [8–10]. The inhibition of the biosynthesis of polar surface lipids such as GPL and PE by NMP and the concomitant decrease in biofilm formation that we report in this study provides a starting point for deeper investigations into the roles of surface lipids in biofilm formation. Further studies are needed to investigate the mechanisms of inhibition of lipid biosynthesis in *M*. *abscessus* by NMP. The incorporation of ^14^C-acetic acid into PE was inhibited by NMP but radiolabel incorporation into CL increased. We did not observe significant effects of NMP on the incorporation of ^14^C-acetic acid into GPL or PI/PIMs (Fig 3A and 3B).

Exponentially-growing *M*. *abscessus* has been shown to accumulate TAG [3, 54]. The accumulation of TAG in *M*. *abscessus* was also shown to increase with its persistence in the human lung [55]. The effects of NMP on ^14^C-palmitate or ^14^C-acetate incorporation into TAG have not been reported previously. We used ^14^C-acetic acid to metabolically radiolabel lipids during log-phase and planktonic growth since it is a precursor of fatty acids synthesized endogenously within the cell. The incorporation of ^14^C-palmitate as well as ^14^C-acetate into the neutral storage lipid TAG in the cellular lipid fraction was significantly increased by NMP in both log-phase and biofilm cells (Fig 4A and 4B). Thus, NMP diverts the metabolic utilization of fatty acids away from the biosynthesis of polar lipids such as PE and CL and towards their utilization in the biosynthesis of the neutral storage lipid TAG in *M*. *abscessus*.

To our knowledge, our findings on the inhibition of efflux pump activity in *M*. *abscessus* biofilms by NMP and PLU (Fig 5B and 5C) have not been reported previously. We found that there was no significant difference in EtBr accumulation at the two temperatures tested (25°C and 37°C; S1 Fig). We observed that glucose did not have a significant effect on the efflux of EtBr in *M*. *abscessus* (S2 Fig). This finding is in agreement with an earlier report which showed that glucose did not have a significant effect on the EtBr efflux activity in *M*. *smegmatis* and *M*. *avium* [56].

Our primary findings on the effects of NMP on lipid biosynthesis in *M*. *abscessus* biofilms are strongly supported by visual data shown in the autoradiograms of the silica-thin-layer chromatography plates (Figs 2–4). A limitation of this study is that our findings were not visualized using confocal or electron micrography of the biofilms. We believe that, for interpreting the effects of NMP on lipid biosynthesis in *M*. *abscessus* biofilms, confocal/ electron micrographs of biofilm ultrastructure will be peripheral to the lipid metabolism data shown, and are outside the scope of this study.

In conclusion, this is the first report showing that NMP inhibits the utilization of fatty acids in the biosynthesis of cell surface GPL in log-phase *M*. *abscessus* and in the biosynthesis of the polar lipids PE and CL in *M*. *abscessus* cells in log-phase and in biofilms. NMP increases the utilization of fatty acids in the biosynthesis of the neutral cellular storage lipid TAG. Our study provides novel findings on the inhibitory effects of NMP and PLU on biofilm formation, lipid biosynthesis, efflux pump activity and antibiotic tolerance all of which are important for pathogenesis by *M*. *abscessus*.

## Supporting information

S1 Fig Impact of temperature on efflux pump inhibitors in mediating ethidium bromide accumulation in *Mycobacterium abscessus*. Effect of temperature on the accumulation of ethidium bromide by *M*. *abscessus* cells in the presence of NMP (A) and PLU (B). After preloading, *M*. *abscessus* cells were washed and re-suspended in PBS solution before exposure to the EPIs. Then the cells were incubated at 25°C and 37°C. Intracellular EtBr fluorescence levels with time are shown. No statistically significant differences observed. Values are expressed as averages from triplicates in a representative experiment.
(TIF)

S2 Fig Effect of glucose and efflux pump inhibitors on the efflux of ethidium bromide by *Mycobacterium abscessus*. *M*. *abscessus* cells in the presence or absence of glucose were treated with NMP (A) and PLU (B) as described in Methods. Intracellular EtBr fluorescence levels with time are shown. Control cells were treated with DMSO. The cells were pre-loaded with 4 μg/mL EtBr in the presence of verapamil at 0.5X MIC. After EtBr-loading, *M*. *abscessus* cells were washed and re-suspended in PBS solution before exposure to the EPIs in the presence or absence of glucose (2%, w/v). No statistically significant differences observed. Values are expressed as averages from triplicates in a representative experiment.
(TIF)

S1 Raw image **Fig 2A.** Digital scan of autoradiogram of TLC plate. ^14^C-Palmitate incorporation into lipids. Cell surface and cellular lipids from log-phase and 3-day biofilm cells of *M*. *abscessus* metabolically radiolabeled with ^14^C-palmitate for 6 h in the presence or absence of NMP were analyzed by silica-TLC using chloroform:methanol:water (65:25:4, v/v/v) as solvent system. Autoradiogram of TLC plate from a representative experiment shown. Equal amounts of total lipid radioactivity loaded in each lane. Three independent experiments performed. Red boxes indicate lipids that showed significant changes upon NMP treatment across experiments. SF, solvent front; GPL, glycopeptidolipids (R_f_ ~0.84); PE, phosphatidylethanolamine (R_f_ ~0.48); CL, cardiolipin (R_f_ ~0.36); PI, phosphatidylinositol; PIMs, phosphatidylinositol mannosides (R_f_ ~0.26–0.1); Ori, origin/ sample loading zone. **Fig 3A.** Digital scans of autoradiograms of TLC plates. ^14^C-Acetate incorporation into surface and cellular lipids from log-phase and 3-day biofilm cells of *M*. *abscessus* metabolically radiolabeled with ^14^C-acetate in the presence or absence of NMP. Autoradiogram of TLC plate from a representative experiment shown. Equal amounts of total lipid radioactivity loaded in each lane. Three independent experiments performed. Cell surface and cellular lipids of log-phase and biofilm cells of *M*. *abscessus* were extracted and analyzed by silica-TLC using chloroform:methanol:water (65:25:4, v/v/v) as solvent system. SF, solvent front; GPL, glycopeptidolipids; PE, phosphatidylethanolamine; CL, cardiolipin; PI, phosphatidylinositol; PIMs, phosphatidylinositol mannosides; Ori, origin/ sample loading zone. **Fig 4A.** Digital scans of autoradiograms of TLC plates. Metabolic radiolabeling of the neutral storage lipid triacylglycerol (TAG) in the cellular lipids of log-phase and 3-day biofilm cells of *M*. *abscessus* with ^14^C-palmitate for 6 h or ^14^C-acetate for 24 h in the presence or absence of NMP was analyzed by silica-TLC using hexane:diethylether:acetic acid (80:20:2, v/v/v). Autoradiogram of TLC plate from a representative experiment shown. Equal amounts of total lipid radioactivity loaded in each lane. SF, solvent front; TAG, triacylglycerol (R_f_ ~0.36); FA, fatty acid (R_f_ ~0.13); Ori, origin/ sample loading zone.
(PDF)

S1 Data Raw data used to generate the graphs shown in Figs 1B, 1E, 2B, 3B, 4B and 5A–5C of representative experiments from three independent biological repeats. (XLSX)
