# Supplementary figures and images for: Biofilm formation and polar lipid biosynthesis in Mycobacterium abscessus are inhibited by naphthylmethylpiperazine
Source: PLoS One. 2024 Nov 12;19(11):e0311669. doi: 10.1371/journal.pone.0311669 (PMC11556751; doi:10.1371/journal.pone.0311669)

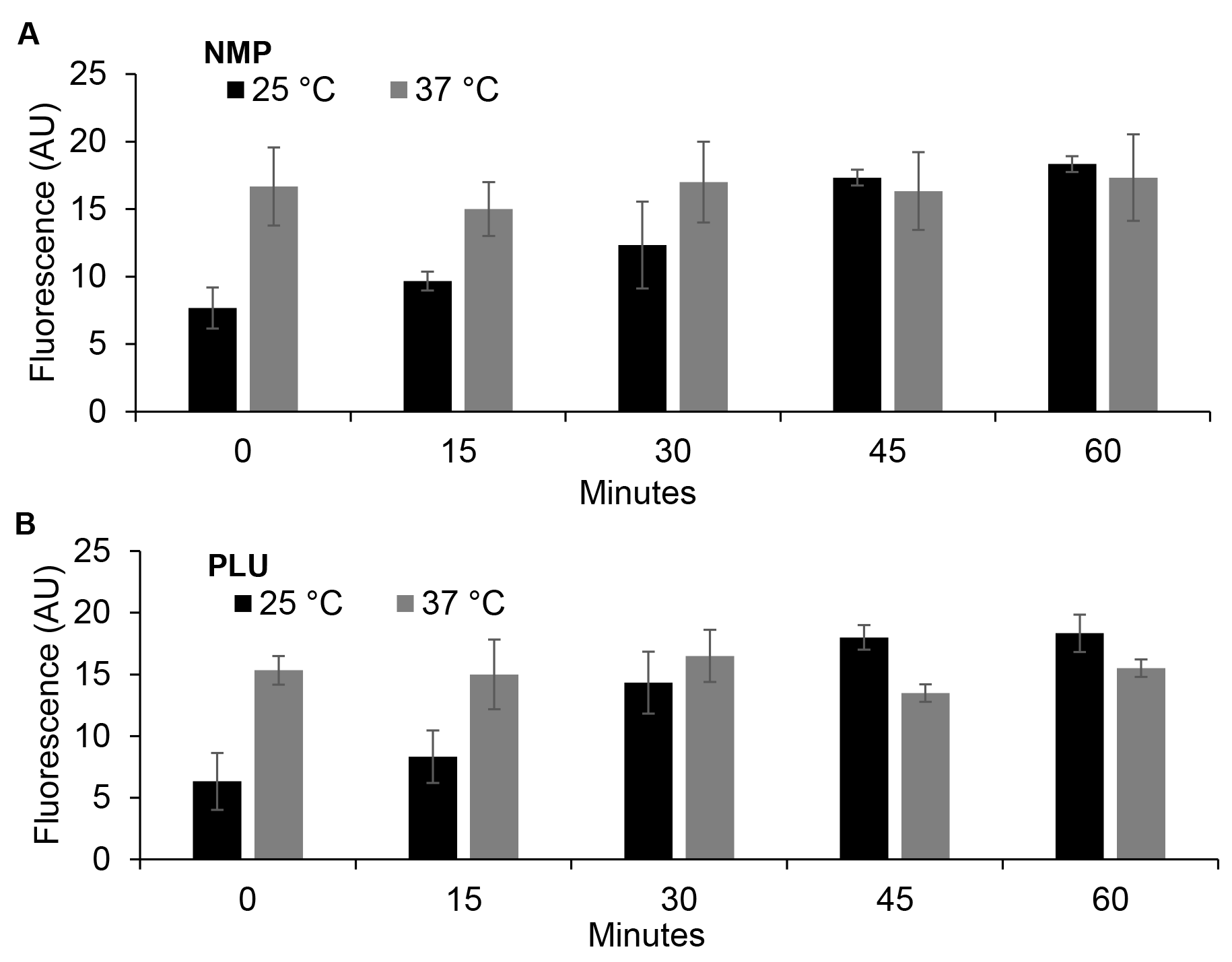

Supplement: S1 Fig — Effect of temperature on the accumulation of ethidium bromide by M. abscessus cells in the presence of NMP (A) and PLU (B). After preloading, M. abscessus cells were washed and re-suspended in PBS solution before exposure to the EPIs. Then the cells were incubated at 25°C and 37°C. Intracellular EtBr fluorescence levels with time are shown. No statistically significant differences observed. Values are expressed as averages from triplicates in a representative experiment. (TIF) [file pone.0311669.s001.tif]

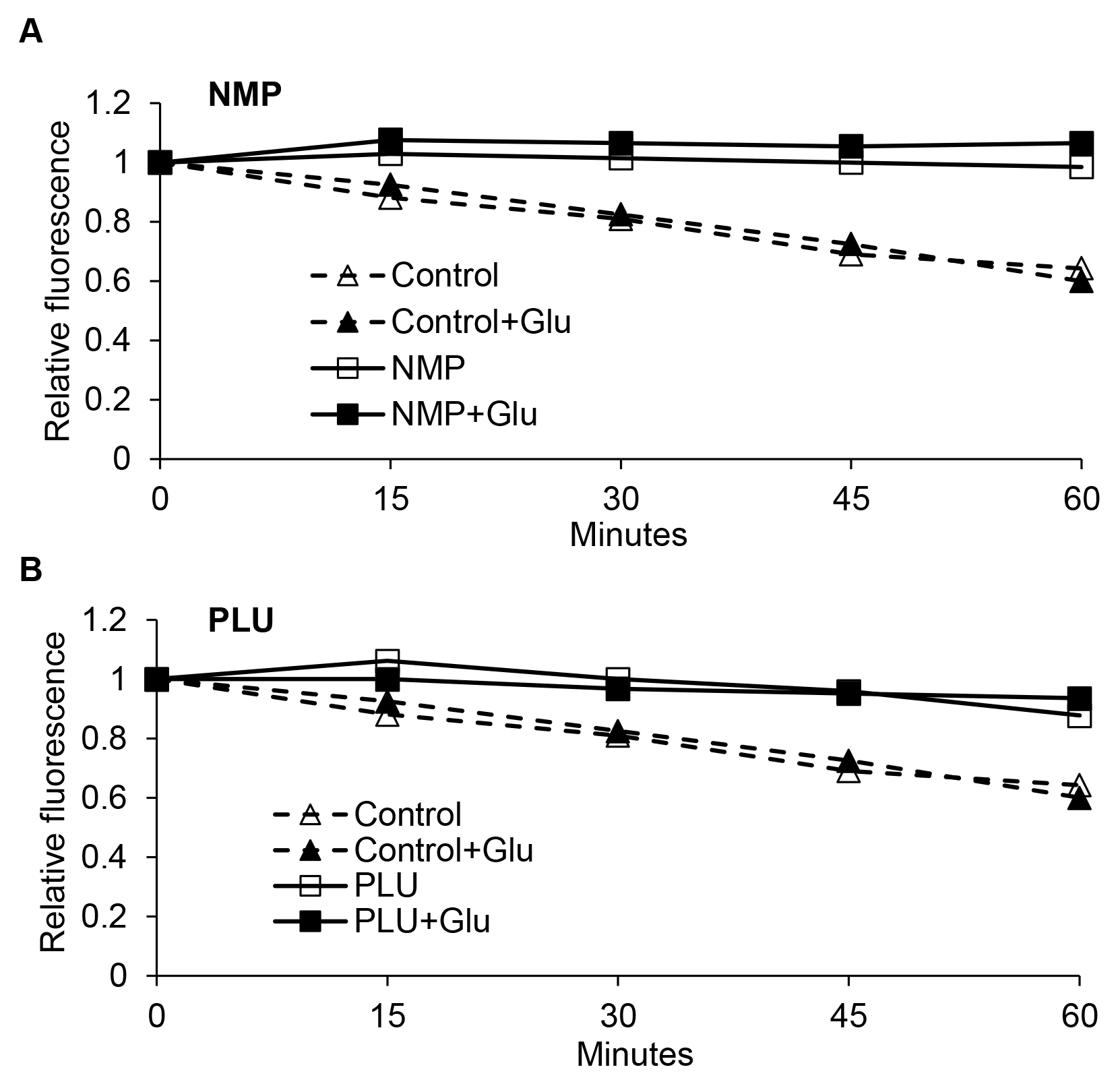

Supplement: S2 Fig — M. abscessus cells in the presence or absence of glucose were treated with NMP (A) and PLU (B) as described in Methods. Intracellular EtBr fluorescence levels with time are shown. Control cells were treated with DMSO. The cells were pre-loaded with 4 μg/mL EtBr in the presence of verapamil at 0.5X MIC. After EtBr-loading, M. abscessus cells were washed and re-suspended in PBS solution before exposure to the EPIs in the presence or absence of glucose (2%, w/v). No statistically significant differences observed. Values are expressed as averages from triplicates in a representative experiment. (TIF) [file pone.0311669.s002.tif]
