# Supplementary material for: Biofilm formation and polar lipid biosynthesis in Mycobacterium abscessus are inhibited by naphthylmethylpiperazine
Source: PLoS One. 2024 Nov 12;19(11):e0311669. doi: 10.1371/journal.pone.0311669 (PMC11556751; doi:10.1371/journal.pone.0311669)

Fig 2A. Digital scan of autoradiogram of TLC plate.
14C-Palmitate incorporation into Lipids

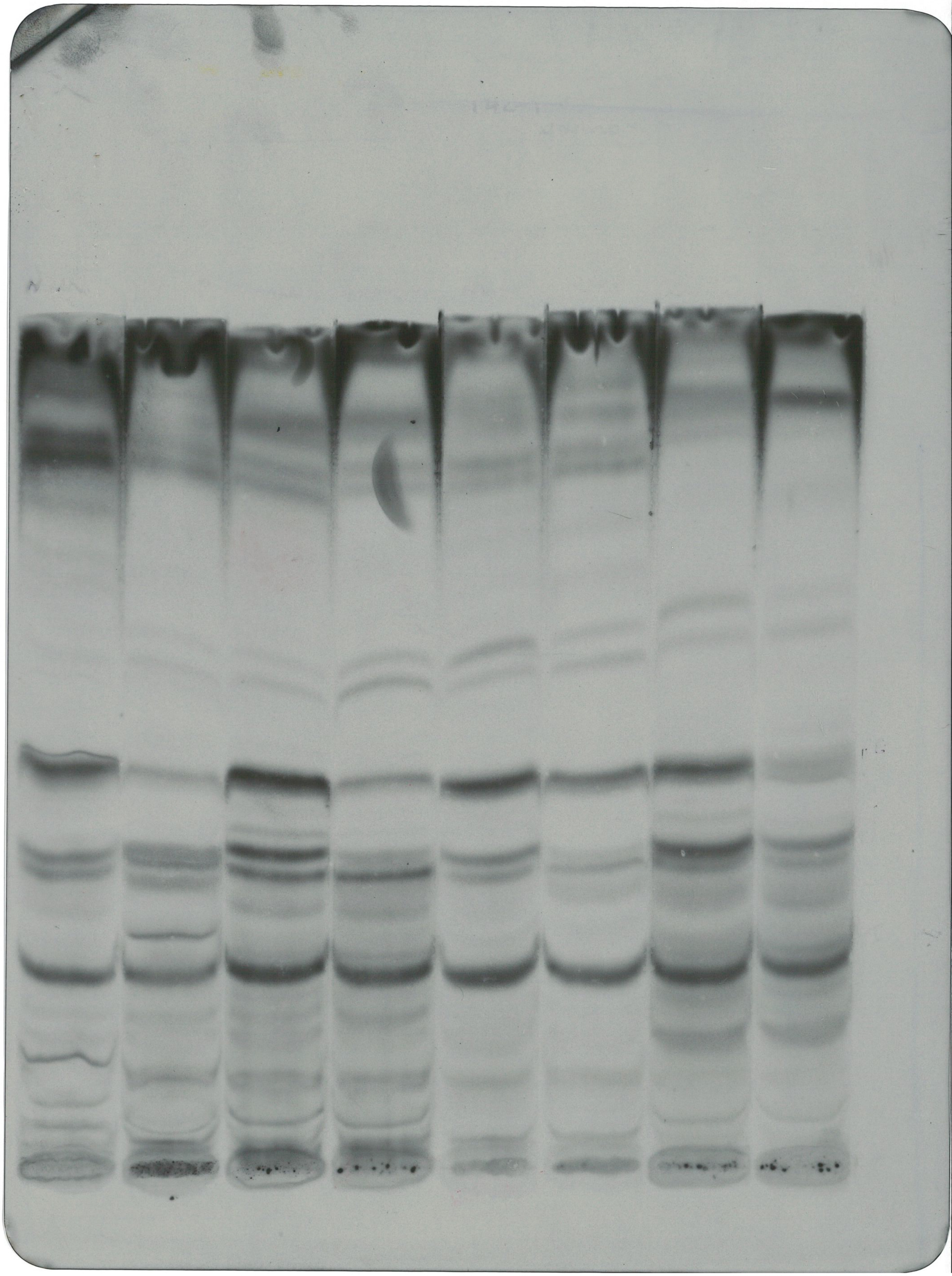

Fig 3A. Digital scan of autoradiogram of TLC plate.
14C-Acetate incorporation into lipids.
Log-phase
Surface Lipids

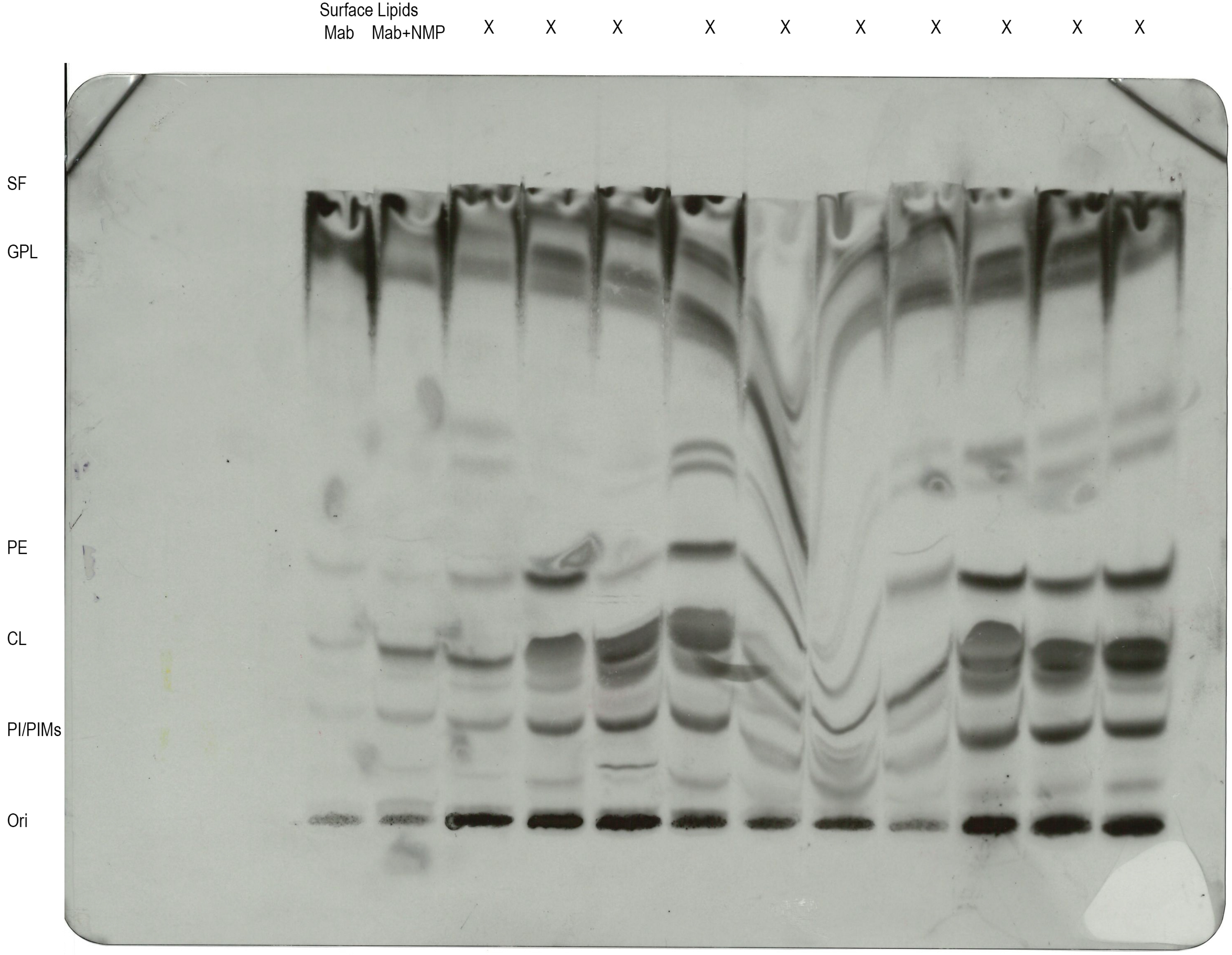

Fig 3A. Digital scan of autoradiogram of TLC plate
14C-Acetate incorporation into lipids
3-day biofilm
Surface lipids

Mab Mab+NMP X X X X X X

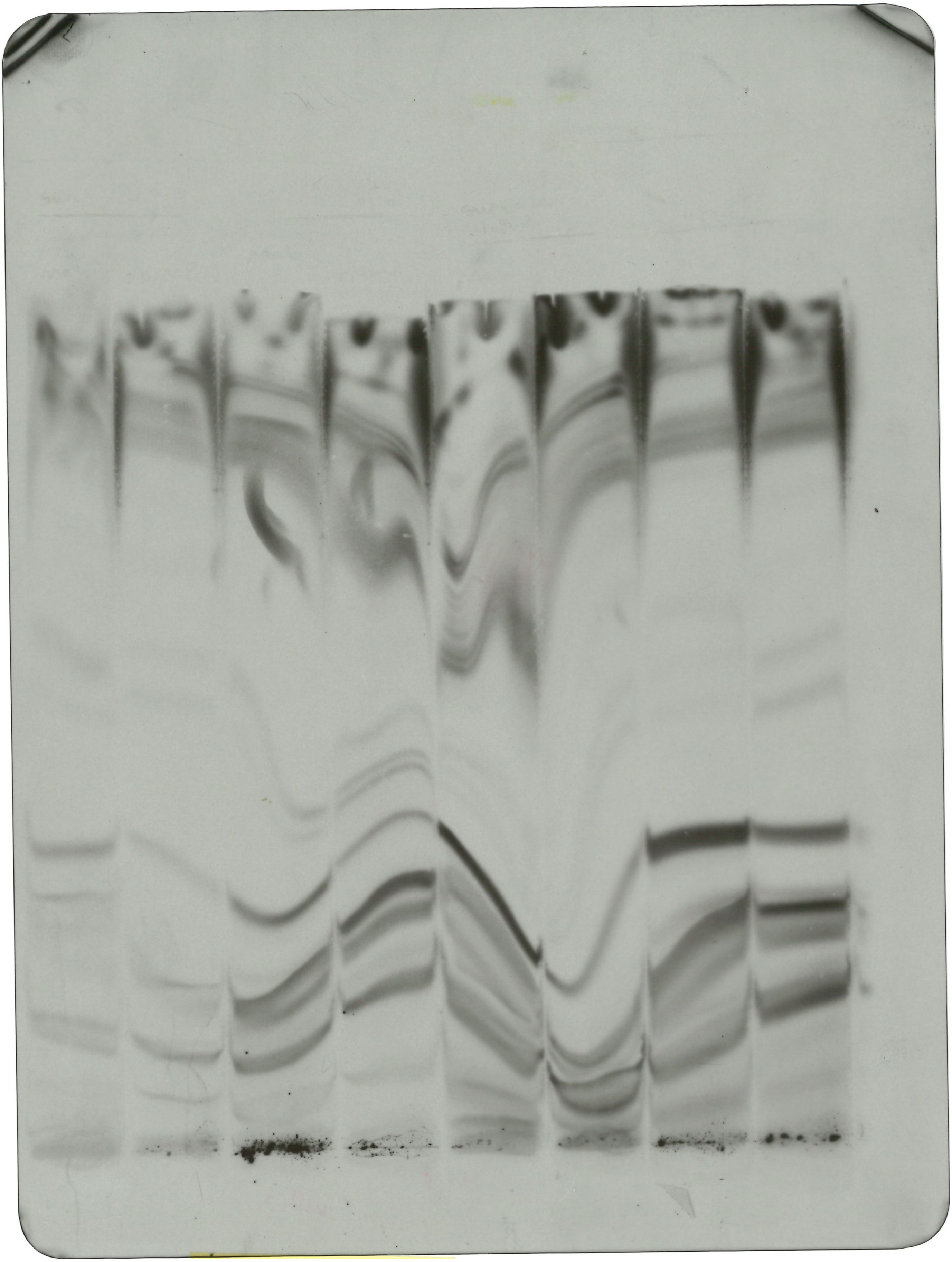

SF
GPL
PE
CL
PI/PIMs
Ori

Fig 3A. Digital scan of autoradiogram of TLC plate.
14C-Acetate incorporation into Cellular lipids

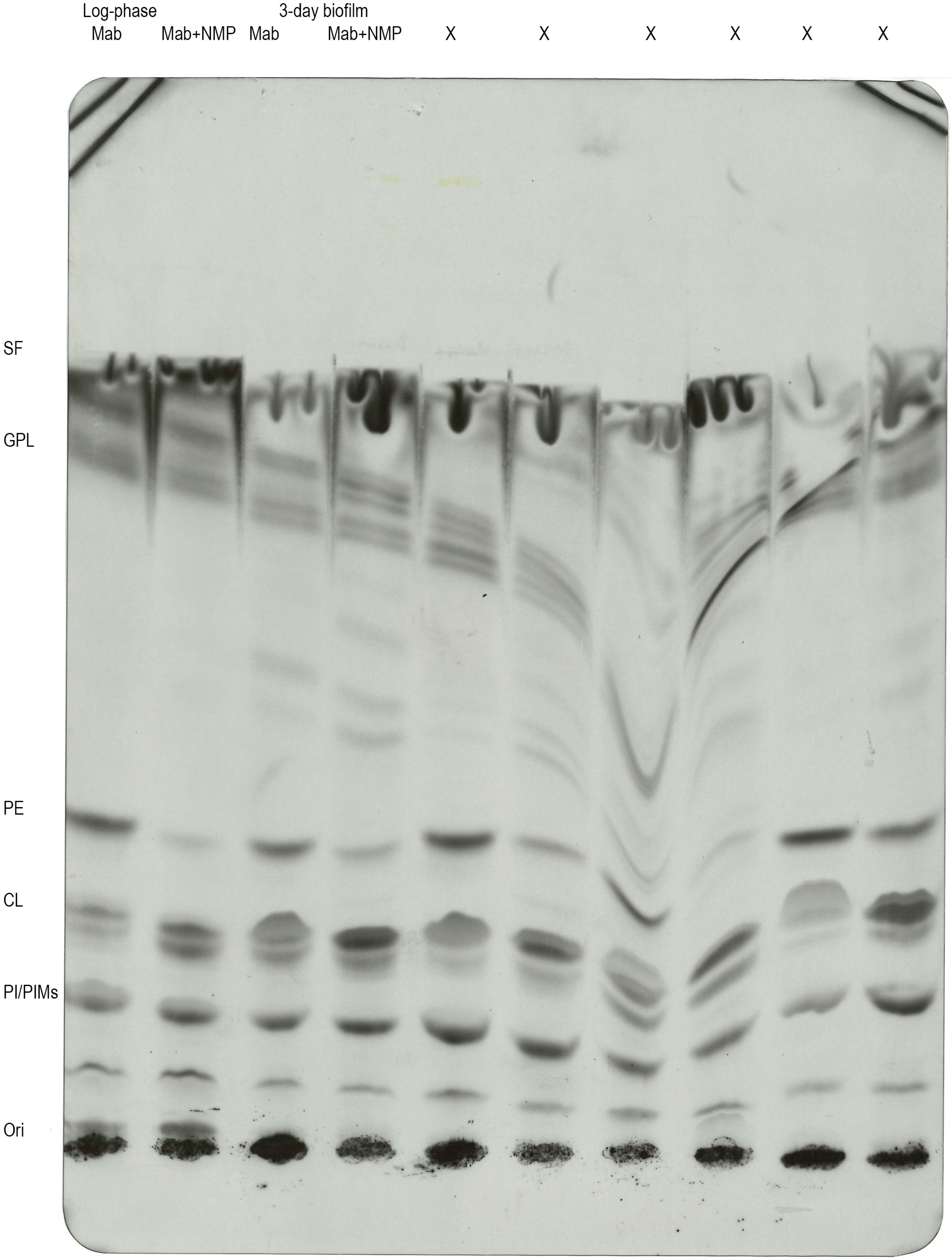

Fig 4A. Digital Scan of autoradiogram of TLC plate.
14C-Palmitate incorporation into Cellular Lipids

Log-phase 3-day biofilm X X X X X X
Mab Mab+NMP Mab Mab+NMP

SF
TAG
FA
Ori

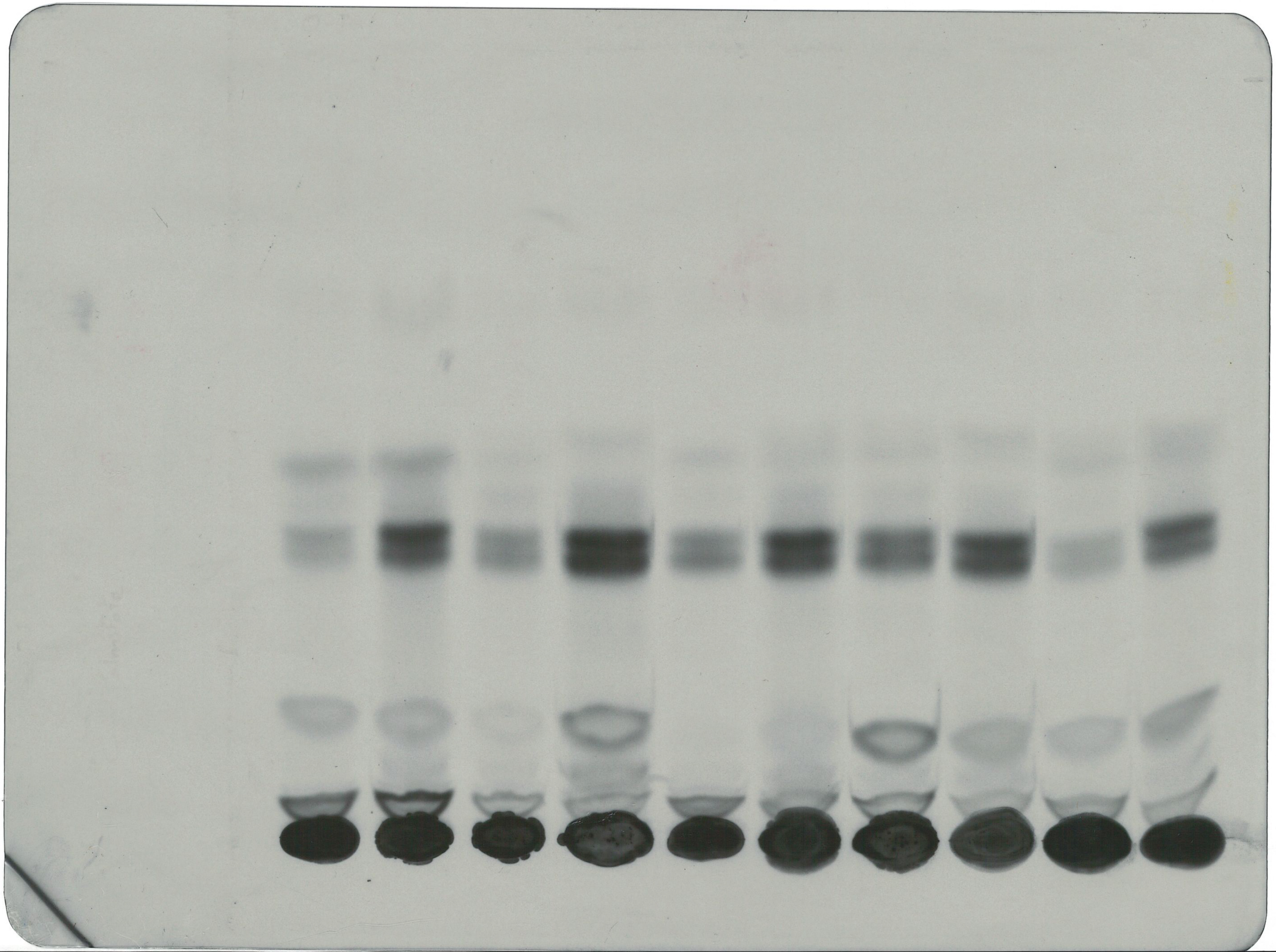

Fig 4A. Digital scan of autoradiogram of TLC plate
14C-Acetate incorporation into Cellular Lipids

Log-phase 3-day biofilm

Mab Mab+NMP Mab Mab+NMP X X X X X X

SF

TAG

FA

Ori

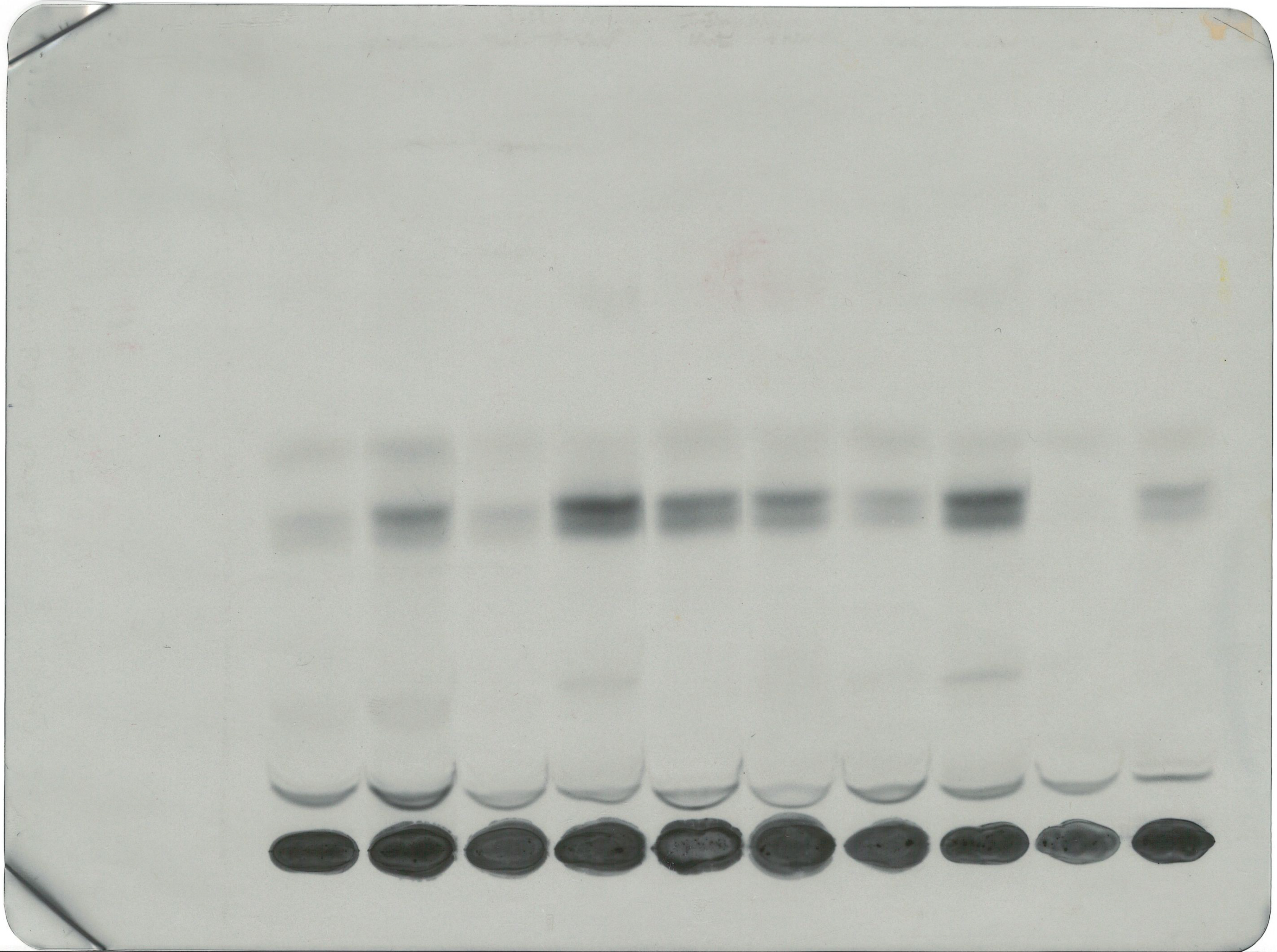

Supplement: S1 Raw image — Fig 2A. Digital scan of autoradiogram of TLC plate. 14C-Palmitate incorporation into lipids. Cell surface and cellular lipids from log-phase and 3-day biofilm cells of M. abscessus metabolically radiolabeled with 14C-palmitate for 6 h in the presence or absence of NMP were analyzed by silica-TLC using chloroform:methanol:water (65:25:4, v/v/v) as solvent system. Autoradiogram of TLC plate from a representative experiment shown. Equal amounts of total lipid radioactivity loaded in each lane. Three independent experiments performed. Red boxes indicate lipids that showed significant changes upon NMP treatment across experiments. SF, solvent front; GPL, glycopeptidolipids (Rf ~0.84); PE, phosphatidylethanolamine (Rf ~0.48); CL, cardiolipin (Rf ~0.36); PI, phosphatidylinositol; PIMs, phosphatidylinositol mannosides (Rf ~0.26–0.1); Ori, origin/ sample loading zone. Fig 3A. Digital scans of autoradiograms of TLC plates. 14C-Acetate incorporation into surface and cellular lipids from log-phase and 3-day biofilm cells of M. abscessus metabolically radiolabeled with 14C-acetate in the presence or absence of NMP. Autoradiogram of TLC plate from a representative experiment shown. Equal amounts of total lipid radioactivity loaded in each lane. Three independent experiments performed. Cell surface and cellular lipids of log-phase and biofilm cells of M. abscessus were extracted and analyzed by silica-TLC using chloroform:methanol:water (65:25:4, v/v/v) as solvent system. SF, solvent front; GPL, glycopeptidolipids; PE, phosphatidylethanolamine; CL, cardiolipin; PI, phosphatidylinositol; PIMs, phosphatidylinositol mannosides; Ori, origin/ sample loading zone. Fig 4A. Digital scans of autoradiograms of TLC plates. Metabolic radiolabeling of the neutral storage lipid triacylglycerol (TAG) in the cellular lipids of log-phase and 3-day biofilm cells of M. abscessus with 14C-palmitate for 6 h or 14C-acetate for 24 h in the presence or absence of NMP was analyzed by silica-TLC using hexa [file pone.0311669.s003.pdf]
